# Gut microbiome shifts in chronic systolic heart failure are associated with disease severity and clinical improvement

**DOI:** 10.1038/s44161-026-00854-y

**Published:** 2026-09-24

**Authors:** Petra Mamic, Handuo Shi, Wenyu Zhou, Nasim Bararpour, Kevin Contrepois, Heyjun Park, Monika Avina, Sophia Miryam Schüssler-Fiorenza Rose, Andrew W. Brooks, Masakazu Kotaka, Paul A. Heidenreich, Kiran Kaur Khush, Michael B. Fowler, W. H. Wilson Tang, Karim Sallam, Justin L. Sonnenburg, Kerwyn Casey Huang, Michael P. Snyder

**Affiliations:** 1 Division of Cardiovascular Medicine, Department of Medicine, Stanford University School of Medicine, Stanford, CA USA; 2 Department of Genetics, Stanford University School of Medicine, Stanford, CA USA; 3 Stanford Cardiovascular Institute, Stanford, CA USA; 4 Department of Microbiology and Immunology, Stanford University School of Medicine, Stanford, CA USA; 5 Department of Bioengineering, Stanford University School of Medicine, Stanford, CA USA; 6 Department of International Health, Johns Hopkins Bloomberg School of Public Health, Baltimore, MD USA; 7 Spinal Cord Injury Service, New Mexico Veterans Affairs Health Care System, Albuquerque, NM USA; 8 Biophysics Program, Stanford University School of Medicine, Stanford, CA USA; 9 Palo Alto Veterans Affairs Health Care System, Palo Alto, CA USA; 10 Kaufman Center for Heart Failure Treatment and Recovery, Heart, Vascular and Thoracic Institute, Cleveland Clinic, Cleveland, OH USA; 11 Department of Cardiovascular and Metabolic Sciences, Lerner Research Institute, Cleveland Clinic, Cleveland, OH USA; 12 Chan Zuckerberg Biohub, San Francisco, CA USA; 13 The Center for Human Microbiome Studies, Stanford University School of Medicine, Stanford, CA USA; 14 Present Address: Biological Sciences Division, University of Chicago, Chicago, IL USA

**Keywords:** Heart failure, Microbiome

## Abstract

The contribution of the gut microbiome to chronic heart failure (HF) remains poorly defined. We performed integrated microbiome and host multi-omic profiling of adults with chronic systolic HF due to nonischemic cardiomyopathy (*n* = 59) and healthy participants (*n* = 50), including longitudinal sampling in some cases. Here we show that patients with HF have depletion of anti-inflammatory *Bifidobacterium* and reduced short-chain fatty acid biosynthetic potential, alongside altered microbiome–immune system interactions. Longitudinal analysis revealed that higher *Bifidobacterium* abundance was associated with clinical improvement. Multi-omic integration prioritized indole-3-propionic acid (IPA), a gut metabolite with cardioprotective properties, as a candidate mechanistic link. We confirmed that *Bifidobacterium* species can produce IPA in vitro and circulating IPA levels were associated with milder HF severity. Together, these findings define gut microbiome alterations in chronic systolic HF, identify candidate microbiome biomarkers of disease severity and trajectory, and suggest microbiome-derived metabolites as potential therapeutic targets.

## Abstract (web summary)

Through multi-omic cohort profiling with longitudinal follow-up in a subset of cases, Mamic et al. identify gut microbiome signatures in nonischemic cardiomyopathy-related systolic heart failure, which are associated with disease severity and clinical improvement.

## Main

Chronic heart failure (HF) affects approximately 6.7 million US adults, and its prevalence is expected to increase^1^. Despite therapeutic advances, HF remains associated with substantial morbidity and mortality^1^. Even with appropriate management, HF trajectories vary, and mechanisms driving progression remain incompletely understood, limiting individualized prediction and therapies.

The gut microbiome has extensive links to human health^2^ and has been implicated in HF pathophysiology^3^. Chronic HF is associated with microbiome disturbance, including expansion of potential pathogens at the expense of anti-inflammatory microorganisms^4–8^, alongside functional shifts including reduced short-chain fatty acid (SCFA) biosynthetic pathways^4,5^ and enriched lipopolysaccharide (LPS) biosynthetic pathways^4^. Animal models show the impact of microbial metabolites on HF physiology. For example, SCFAs, byproducts of microbial fermentation of dietary fiber, regulate cardiac remodeling, vascular tone and immune function, and fuel the failing myocardium^3,9,10^. However, mechanistic links between the gut microbiome and human HF pathophysiology remain unclear, as previous studies have often been small and cross-sectional, and lacked functional profiling.

Here we performed integrated gut microbiome and host multi-omic profiling in patients with chronic systolic HF related to nonischemic cardiomyopathy (NICM), including longitudinal sampling and clinical follow-up in a subset of patients. We identified taxonomic and functional gut microbiome signatures associated with HF, as well as microbiome, metabolome and immunome features associated with disease severity and clinical improvement. These findings reveal potential mechanisms through which gut microorganisms may influence host physiology in chronic systolic HF.

## Results

### Cohort description

**Fig. 1:**
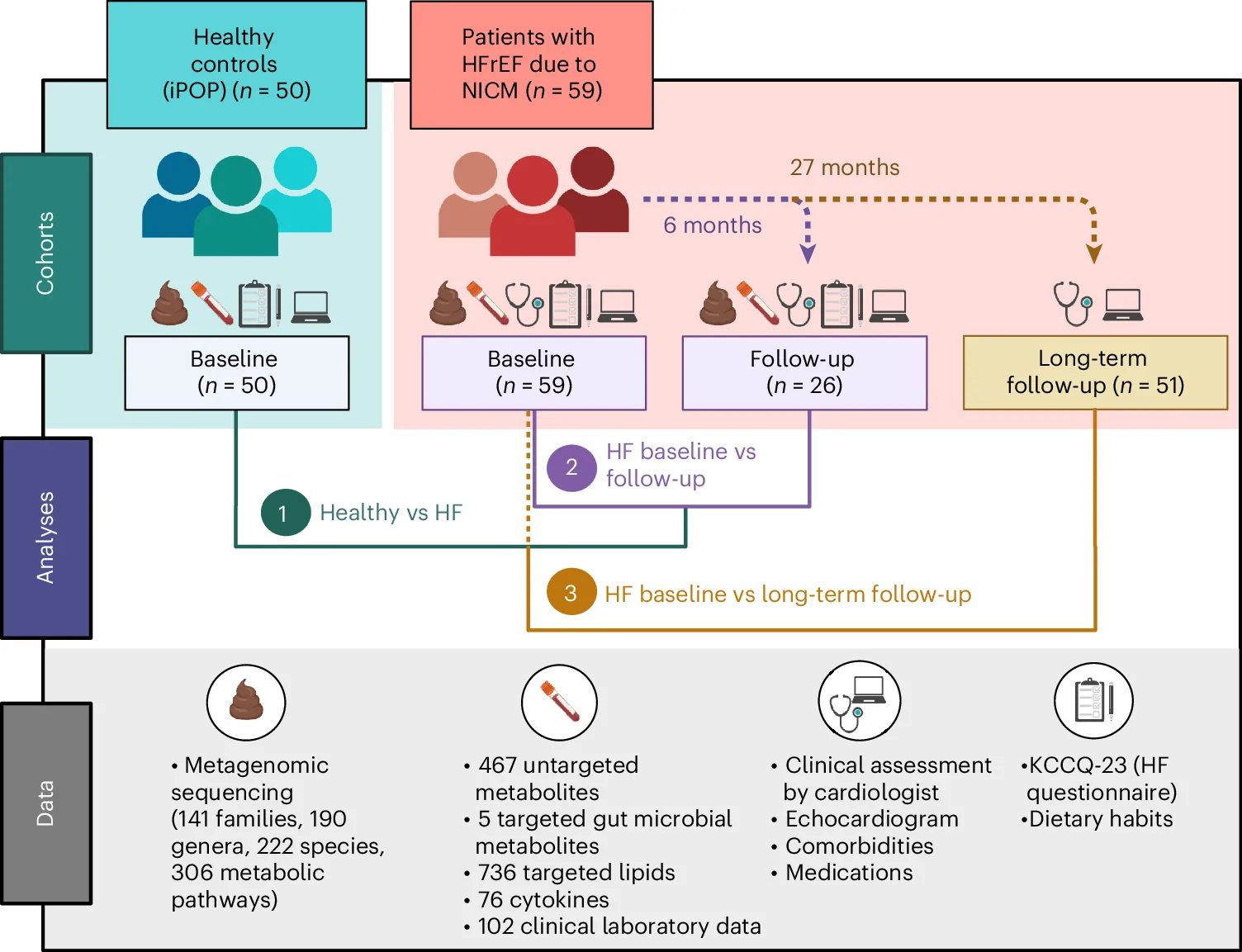
Design of the multi-omic study with longitudinal profiling and clinical phenotyping of a cohort of 59 adults with chronic HF with reduced ejection fraction due to NICM. Adults with stable chronic HF with reduced ejection fraction (HFrEF) due to NICM (*n* = 59) were recruited for the study. During the baseline visit, each participant underwent multi-omic profiling, including whole-genome sequencing of microbial DNA extracted from stool, untargeted metabolomics, targeted lipidomics from host plasma, targeted metabolomics of select gut microbial metabolites and a 76-cytokine Luminex immune panel from host serum, as well as extensive clinical laboratory profiling. All individuals with chronic HF were additionally assessed by their regular HF cardiologist within days of their baseline study visit. Clinical data (patient functional cardiac status and symptomatology, echocardiographic parameters, comorbidities and medications) were extracted from the EMR. Study participants additionally completed questionnaires related to their cardiac functional status and HF symptoms (KCCQ-23) and habitual diet. A follow-up visit was completed by 26 participants after an average of 6 months from their baseline visit, at which time they underwent repeat multi-omic profiling and clinical assessment and completed questionnaires. For 51 of the patients with at least one visit, limited clinical data (clinical status and limited echocardiographic data) were available at an average of 27 months following their baseline visit and were extracted from the EMR. Healthy participants (*n* = 50) were previously recruited for the iPOP study^22^, during which they were profiled extensively on a quarterly basis. Stool and serum samples and questionnaire data from the most recent healthy visit were included for this study. Figure created in BioRender; Mamic, P. https://biorender.com/q09hxwx (2026).

Figure 1 summarizes the study design, data collection and analyses. We recruited 59 adults with chronic systolic HF and 50 healthy controls. Baseline demographics, comorbidities, medications and laboratory values are presented in Table 1. The cohorts were broadly similar in age, body mass index (BMI) and racial composition. Women were underrepresented in the HF cohort (22% versus 52%, *P* = 0.001).

**Table 1:** Baseline characteristics of individuals from the HF and healthy cohorts

	Chronic HF (*n* = 59; mean (s.d.), median [IQR] or %)	Healthy (*n* = 50; mean (s.d.), median [IQR] or %)	*P* value
**Demographics**			
Age (years)	52.3 (10.9)	55.3 (8.7)	0.12
Female sex	22.0%	52.0%	0.001
Race or ethnicity			0.41
White	69.5%	66.0%	
Black or African American	8.5%	8.0%	
East Asian	6.8%	4.0%	
Hispanic	3.3%	6.0%	
South Asian	6.8%	16.0%	
Other	5.1%	0.0%	
BMI (kg m^−2^)	27.4 [25.1, 32.1]	28.1 [25.5, 31.1]	0.69
**Comorbidities**			
Hypertension	16.9%	24.0%	0.47
Hyperlipidemia	32.2%	34.0%	1
Atrial fibrillation	32.2%	0.0%	3.12 × 10^−6^
Ventricular tachycardia	23.7%	0.0%	8.88 × 10^−5^
Chronic kidney disease	22.0%	0.0%	0.0003
GERD	16.9%	16.0%	0.72
**Medications**			
Beta blocker	96.7%	8.0%	3.84 × 10^−26^
ACE-i, ARB or ARNI	93.2%	12.0%	3.31 × 10^−19^
MRA	81.4%	0.0%	1.75 × 10^−20^
Diuretic (loop or thiazide)	52.5%	10.0%	1.78 × 10^−6^
Digoxin	32.2%	0.0%	3.12 × 10^−6^
Statin	22.0%	20.0%	0.82
Proton pump inhibitor	11.9%	14.0%	0.78
Warfarin	10.2%	0.0%	0.03
**Clinical laboratory tests**			
Sodium (mmol l^−1^)	138 [137, 140]	139 [137, 141]	0.13
Creatinine (mg dl^−1^)	1.01 [0.88, 1.25]	0.86 [0.76, 1.0]	0.0001
Total bilirubin (mg dl^−1^)	0.6 [0.5, 0.8]	0.5 [0.4, 0.7]	0.08
Albumin (g dl^−1^)	4.2 (0.4)	3.9 (0.3)	4.81 × 10^−6^
Hemoglobin A1c (%)	5.7 [5.5, 6.1]	5.6 [5.3, 5.7]	0.007
WBC (×1,000, per µl)	6.2 [5.4, 7.0]	5.4 [4.5, 6.3]	0.013

Student’s *t*-tests were used for between-group comparison of normally distributed continuous variables; Mann–Whitney *U* tests were used for continuous variables with skewed distributions; Fisher’s exact tests were used for categorical variables. All statistical tests were two sided.

ACE-i, angiotensin-converting enzyme inhibitor; ARB, angiotensin II receptor blocker; ARNI, angiotensin receptor–neprilysin inhibitor; MRA, mineralocorticoid receptor antagonist; GERD, gastroesophageal reflux disease; IQR, interquartile range; WBC, white blood cell count.

Baseline HF characteristics are summarized in Extended Data Table 1. Participants had a markedly reduced left ventricular ejection fraction (LVEF) and a mildly to moderately dilated left ventricle (LV), and more than half had right ventricular (RV) dysfunction. Nevertheless, the cohort was overall clinically well compensated. Compared with healthy participants, patients with HF had more comorbidities and medications (Table 1 and Supplementary Table 1), notably cardiac medications comprising guideline-directed medical therapy for systolic HF.

A total of 26 patients with HF were resampled after a mean of 6 months. Clinical follow-up data were available for 51 patients at an average of 27 months after the baseline. During follow-up, 12 patients experienced a poor clinical outcome (composite of heart transplant, durable left ventricular assist device (LVAD) implantation, hospice or death); 14 experienced clinical improvement (based on changes in New York Heart Association (NYHA) functional class, LVEF or Kansas City Cardiomyopathy Questionnaire (KCCQ)-23).

### Gut microbiome taxonomic composition differs between patients with chronic HF and healthy participants

**Extended Data Fig. 1:**
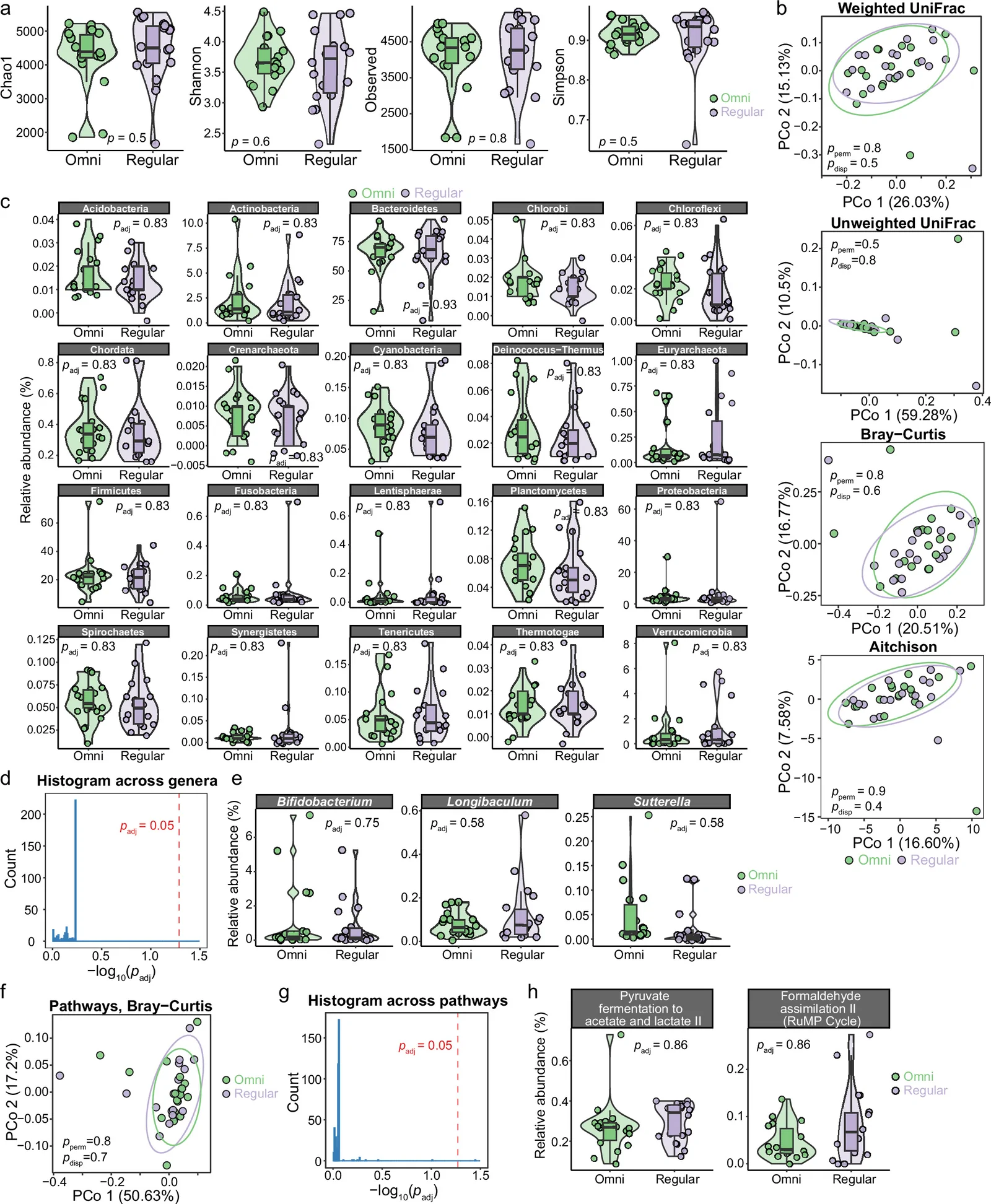
No significant batch effects were observed between fresh frozen stool samples and samples preserved using OMNIgene-GUT collection kit. We collected stool samples using simultaneous direct freezing and OMNIgene-GUT collection kit (*n* = 18, total of 36 samples). **a-d**, Paired samples exhibited no significant differences in alpha- (**a**) or beta-diversity (**b**) at the taxonomic level. We used a univariate linear model to evaluate differences at the phylum (**c**) and genus (**d**) levels, which showed no significant differences. **e**, For select genera that exhibited significant differences between healthy participants and patients with HF (Fig. 2c), the differences due to sample collection methods were smaller in magnitude and not statistically significant. **f**, Similarly, we did not observe any differences in pathway beta-diversity. **g**, When using a univariate linear model to evaluate potential bias from sampling methods in pathway abundances, we found that the teichoic acid (poly-glycerol) biosynthesis pathway (TEICHOICACID-PWY; *p*_adj_ = 0.04) and L-lysine biosynthesis II pathway (PWY-2941; *p*_adj_ = 0.098) were the only two pathways affected by sampling methods (both pathways excluded for downstream analyses). **h**, For select pathways that exhibited significant differences between the two cohorts (Fig. 2e), the differences due to sample collection methods were smaller in magnitude and not statistically significant. In the boxplots, the center line represents the median value, the box limits show the interquartile range (IQR), and the line whiskers extend to 1.5 times the IQR. For beta-diversity analyses, ellipses represent 95% confidence intervals. *p*_adj_, *p*-values after FDR correction using the Benjamini-Hochberg method; *p*_perm_, *p*-values from permutational multivariate analysis of variance (PERMANOVA); *p*_disp_, *p*-values from beta dispersion analyses. Source data

**Extended Data Fig. 2:**
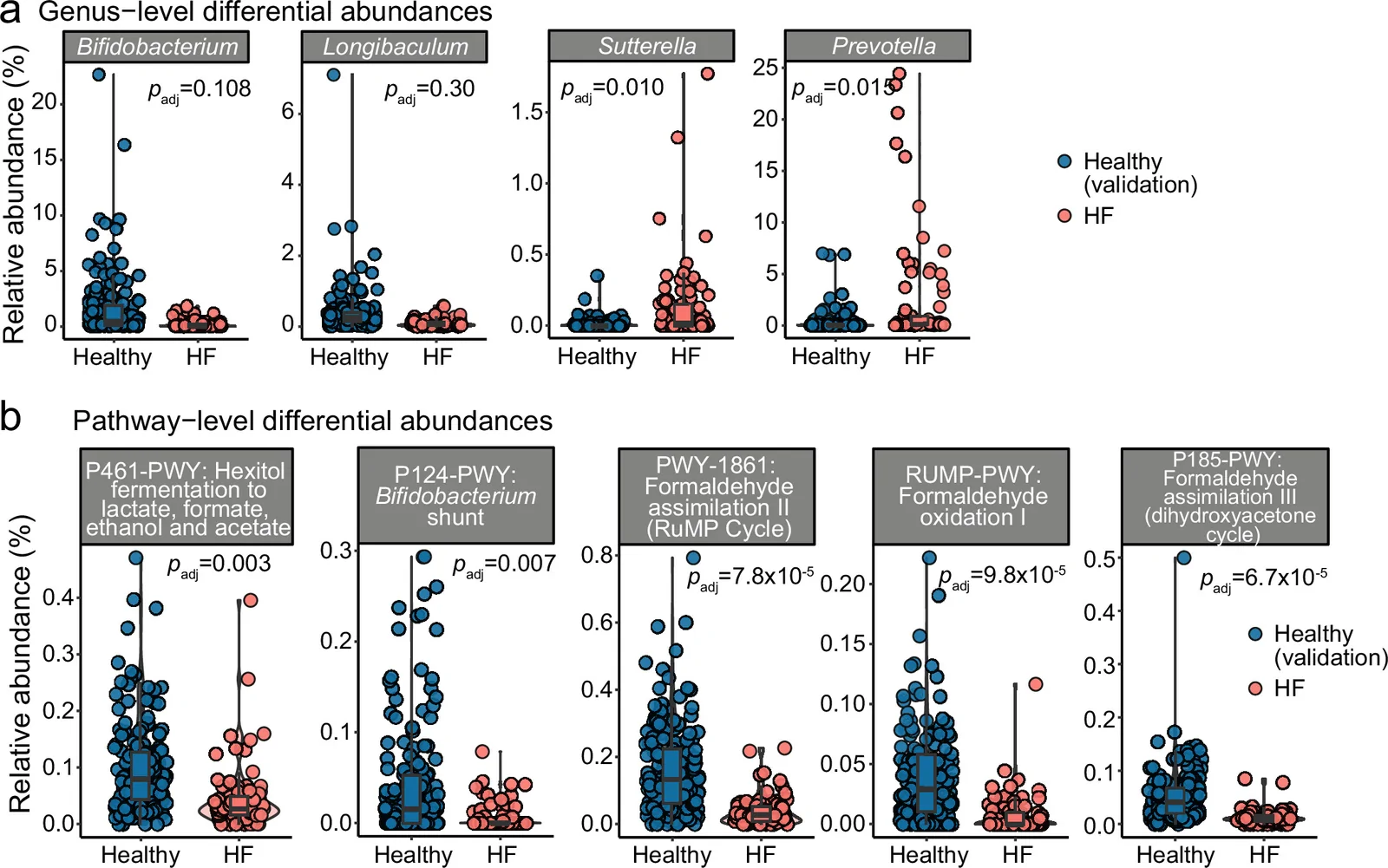
Validation of chronic heart failure-associated gut microbiome signatures using a validation healthy cohort. We validated the key genera (**a**) and pathways (**b**) that were differentially abundant in the heart failure (HF) cohort (*n* = 59 subjects, with total of 86 samples), compared to the additional validation cohort of healthy participants (*n* = 61, with total of 168 samples) with comparable male/female ratio to the HF cohort. Individual genus or pathway was modeled as a dependent continuous variable in a linear mixed-effects model with age, sex, race, body mass index, and categorical HF as fixed effects, and participant as a random effect. Coefficients were tested using two-sided *t*-tests with degrees of freedom calculated using Satterthwaite’s approximation. In the boxplots, the center line represents the median, box limits show the interquartile range (IQR), and whiskers extend to 1.5*IQR. *p*-values were corrected for multiple hypotheses testing using the Benjamini-Hochberg method. Source data

We performed metagenomic sequencing of stool samples to profile gut microbiome composition. Although stool collection methods differed between the HF and healthy cohorts, we found no substantial batch effect (Methods, Extended Data Fig. 1 and Supplementary Table 2). To address the between-cohort sex imbalance, we repeated HF–healthy comparisons using a larger healthy validation cohort (Supplementary Information, Extended Data Fig. 2 and Supplementary Table 3).

**Fig. 2:**
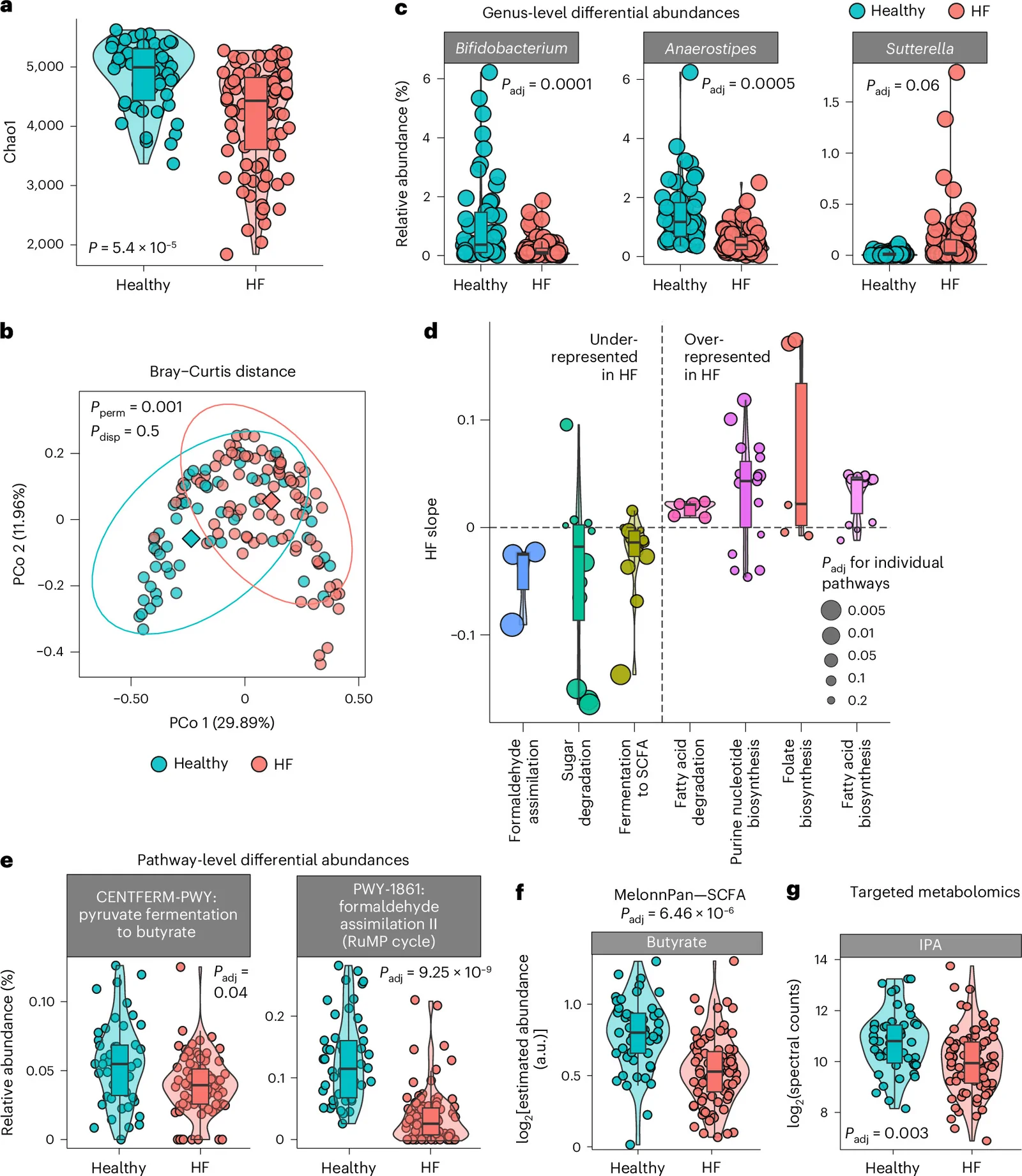
Chronic systolic HF is associated with distinct taxonomic and functional gut microbiome shifts. **a**, Patients with chronic HF (*n* = 59) had overall lower alpha-diversity than the healthy cohort (*n* = 50). **b**, Beta-diversity of taxonomic composition across healthy and HF cohorts, showing distinct compositions (Bray–Curtis distance); ellipses represent 95% confidence intervals. **c**, Representative differentially abundant taxa between the cohorts included *Bifidobacterium* and *Anaerostipes* (depleted in HF), and *Sutterella* (enriched in HF). **d**, GSEA highlighted systematically over- or underrepresented gut microbial pathways in the HF-associated gut microbiome. Key individual pathways are shown in **e**. **f**,Consistent with the GSEA results, MelonnPan predicted decreased gut microbiome-wide output of SCFA butyrate. **g**, Circulating levels of IPA were lower in HF. In **a**–**c** and **e**–**g**, individual microbiome features were modeled as dependent continuous variables in a linear mixed-effects model with age, sex, race, BMI, dietary patterns and categorical HF as fixed effects, and participant as a random effect. Coefficients were tested using two-sided *t*-tests with degrees of freedom calculated using Satterthwaite’s approximation. Each figure included all available data points (baseline and follow-up visits for patients with HF for a total of 86 visits, and a single visit from each healthy participant). For GSEA (**d**), the *P* value of each pathway group was estimated via 1,000 permutations. In boxplots (**a** and **c**–**g**), the center line represents the median, box limits show the IQR and whiskers extend to 1.5 × IQR. To adjust for multiple hypothesis testing, the Benjamini–Hochberg method was used throughout (**a** and **c**–**g**). Chao1, Chao1 index of alpha-diversity; *P*_perm_ and *P*_disp_, *P* values from permutational multivariate analysis of variance (PERMANOVA) testing for significant differences in group centroids and within-group dispersion, respectively; PCo1 and PCo2, principal coordinate axes 1 and 2; RuMP, ribulose monophosphate. Source data

**Extended Data Fig. 3:**
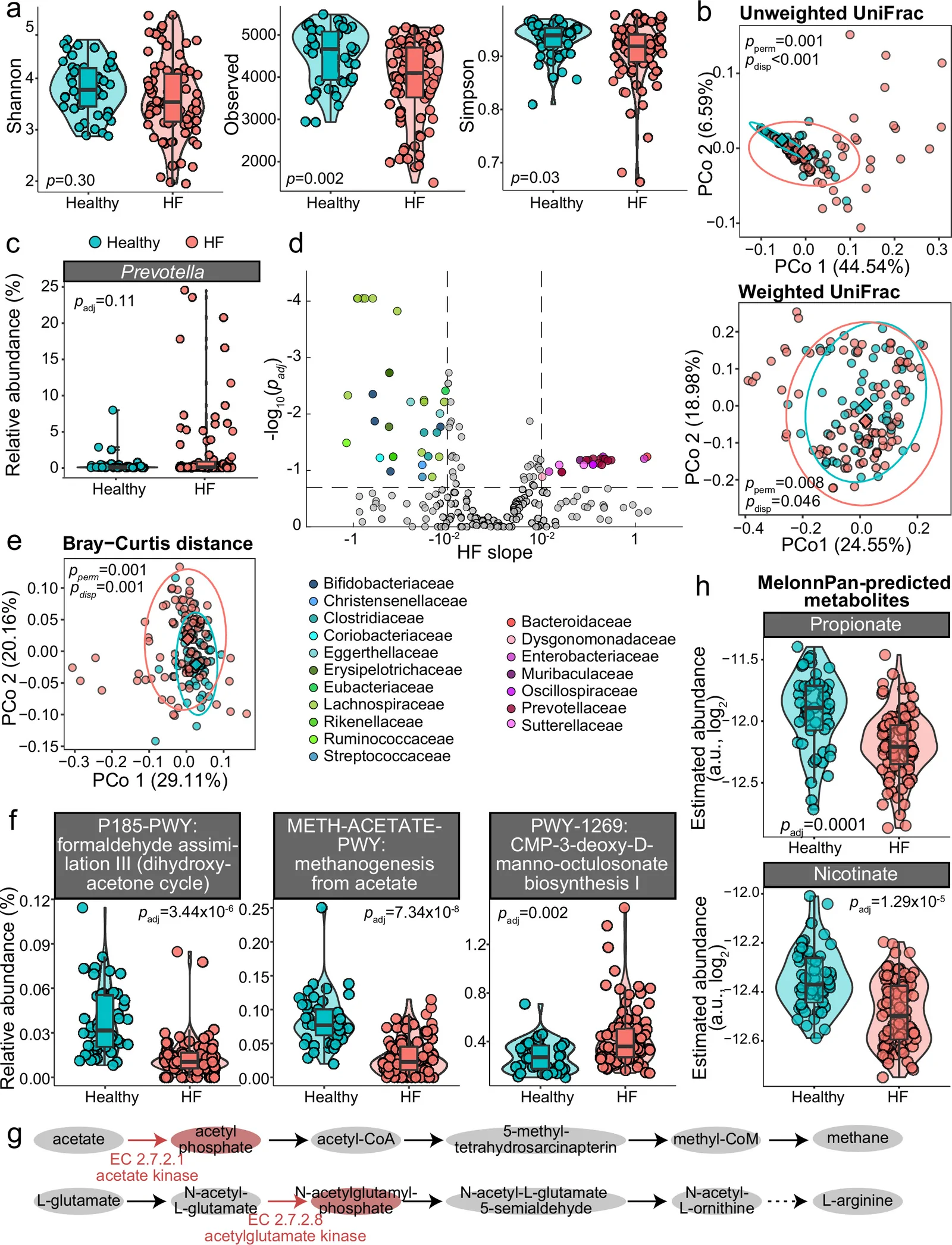
Chronic systolic heart failure is associated with distinct taxonomic and functional gut microbiome changes. **a**, Patients with HF (*n* = 59, with total of 86 samples) had overall lower alpha-diversity compared to the healthy cohort (*n* = 50), as measured by the number of observed species, and Simpson index. There was no significant between-cohort difference in Shannon diversity. **b**, The cohorts had significantly different taxonomic compositions (beta-diversity using unweighted and weighted UniFrac distance). **c**, *Prevotella* was enriched in the HF cohort. **d**, Volcano plot of differentially abundant species in healthy and HF cohorts (*p* < 0.05, *p*_adj_ < 0.2, with absolute slope >0.01. **e**, Pathway-level beta-diversity analysis revealed distinct functional microbiome between-cohort compositions. **f**, Representative pathways differentially abundant in patients with HF. **g**, Flow charts of methanogenesis from acetate (METH-ACETATE-PWY) (top) and L-arginine biosynthesis pathway (bottom), with the rate-limiting enzymes and molecules in patients with HF (red). **h**, MelonnPan predicted a significantly decreased microbiome-wide output of propionate (short chain fatty acid) and nicotinate (vitamin B3). (**a, c-d**, **f**, **h**) Linear mixed-effects models (microbiome features as dependent variables, HF as independent variable) were adjusted for age, sex, race, body mass index, diet, categorical HF (fixed effects), and participant (random effect). Two-sided *t*-tests with Satterthwaite’s degrees of freedom approximation were used. Figures show all data points, including repeated visits for patients with HF. For boxplots, the center line represents the median, box limits show the interquartile range (IQR), and whiskers extend to 1.5*IQR. In beta-diversity analyses, ellipses represent 95% confidence intervals. Benjamini-Hochberg method was used to adjust for multiplicity. *p*_perm_, *p*-values from permutational multivariate analysis of variance (PERMANOVA); *p*_disp_, *p*-values from beta dispersion analyses. Source data

Gut microbiome alpha-diversity was lower in patients with chronic HF (Fig. 2a and Extended Data Fig. 3a), and beta-diversity differed globally between the cohorts (*P* = 0.001, permutational multivariate analysis of variance (PERMANOVA) test; Fig. 2b and Extended Data Fig. 3b). Differential abundance analysis identified HF-associated depletion of families Lachnospiraceae and Bifidobacteriaceae, and genera *Bifidobacterium*, *Anaerostipes*, *Anaerobutyricum*, *Lachnospira* and *Blautia* (*P*_adj_ < 0.001 for all, where *P*_adj_ refers to the Benjamini–Hochberg correction for multiple hypotheses testing; Fig. 2c and Supplementary Table 4). These taxa include microorganisms associated with SCFA production from available precursors such as dietary fiber and host anti-inflammatory effects^11^. *Sutterella* (*P*_adj_ = 0.064; Fig. 2c) and *Prevotella* (*P*_adj_ = 0.114; Extended Data Fig. 3c), linked to pro-inflammatory host responses^12^, were enriched in HF. Most of the >80 differentially abundant species were depleted in HF (Extended Data Fig. 3d and Supplementary Table 4). Together, these findings indicate that chronic HF is associated with loss of SCFA-associated and anti-inflammatory taxa, and enrichment of pro-inflammatory microorganisms. While this may suggest an overall pro-inflammatory shift, the observed effects may be influenced by dietary factors such as fiber intake, which affects SCFA production.

### Functional analyses indicate reduced SCFA biosynthetic potential in chronic HF

Gut microbial pathway composition was notably different between cohorts (*P* = 0.001, PERMANOVA test; Methods, Extended Data Fig. 3e and Supplementary Table 4). Pathways involved in SCFA biosynthesis and formaldehyde detoxification were depleted in HF (Fig. 2d,e and Extended Data Fig. 3f), as were methanogenesis from acetate and l-arginine biosynthesis pathways (Extended Data Fig. 3f,g). By contrast, pathways related to biosynthesis of LPS, a pro-inflammatory component of the outer membrane in Gram-negative microorganisms^13^, were enriched in HF (Extended Data Fig. 3f).

Systematic identification of HF-associated functional groups using gene set enrichment analysis (GSEA; Methods) confirmed broad depletion of fermentative and formaldehyde detoxification pathways, along with enrichment of lipid metabolism, folate and purine nucleotide biosynthetic pathways (Fig. 2d and Supplementary Table 4). These findings are consistent with a systematic shift from anaerobic carbohydrate fermentation to oxidative lipid metabolism, previously also associated with other chronic diseases^14,15^. Consistent with these results, metagenomics-predicted luminal levels of SCFAs butyrate (Fig. 2f) and propionate (Extended Data Fig. 3h) were lower in HF (Supplementary Table 4). Altogether, our results suggest profound microbiome functional shifts in chronic HF that may have systemic host effects.

### Circulating indole-3-propionic acid is reduced in chronic HF

To assess how gut microbiota changes affected systemic levels of microbiome-derived metabolites, we measured circulating SCFAs, indole-3-propionic acid (IPA) and indole-3-lactic acid (ILA) using targeted metabolomics (Supplementary Table 4). While circulating SCFAs and ILA did not differ between cohorts, IPA, a microbial derivative of dietary tryptophan with cardioprotective properties^16–19^, was markedly reduced in HF (Fig. 2g), suggesting altered microbial tryptophan metabolism with potential implications for HF pathophysiology.

### Chronic HF alters patterns of gut microbiome–immune system interactions

**Extended Data Fig. 4:**
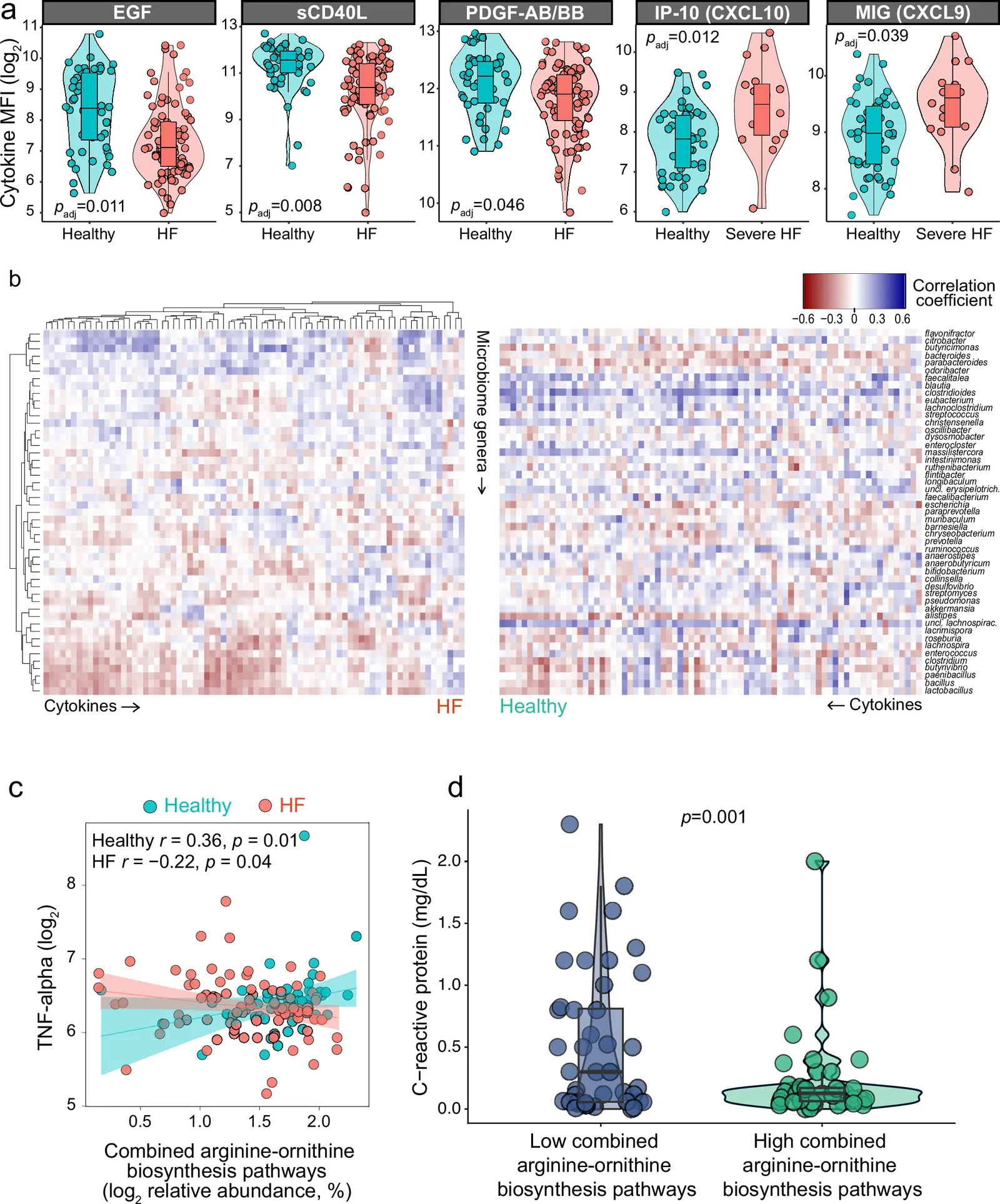
Individual cytokines were differentially abundant in patients with chronic HF compared to healthy participants. **a**, Differentially abundant cytokines between chronic HF (*n* = 58) and healthy cohorts (*n* = 48), as well as between patients with more severe HF (New York Heart Association class III-IV, *n* = 14) and healthy. Linear mixed-effects model accounted for age, sex, race, body mass index (fixed effects) and participant (random effect). Benjamini-Hochberg method was used to adjust for multiplicity. **b**, Correlation heatmap between the top 50 most abundant microbial genera (vertical axis) and 76 cytokines (horizontal axis). Hierarchical clustering was performed using pairwise Spearman correlations (on both microbiome and cytokine levels) for the HF cohort (*n* = 58, left), with this cluster structure applied to microbiome-cytokine correlations in the healthy cohort (*n* = 48, right). **c**, The microbiome pathway cluster that showed the most divergent and cohort-specific cytokine correlations includes arginine and ornithine biosynthesis pathways (ARGSYN-PWY: L-arginine biosynthesis I, ARGSYNBSUB-PWY: L-arginine biosynthesis II (acetyl cycle), PWY-5154: L-arginine biosynthesis III via N-acetyl-L-citrulline, PWY-7400: L-arginine biosynthesis IV (archaebacteria), and GLUTORN-PWY: L-ornithine biosynthesis I) (black box in Fig. 3a). Their aggregate relative abundance showed cohort-specific association with pro-inflammatory cytokine TNF (**c**). Aggregated pathways were log_2_-transformed for visualization purposes. In the HF cohort, low arginine/ornithine biosynthetic pathway group (defined as aggregate relative abundance below median) had significantly higher C-reactive protein, a clinical biomarker of systemic inflammation (**d**). In boxplots, the center represents the median, box limits show the interquartile range (IQR), with whiskers extending to 1.5*IQR. Scatter plots show linear fit (solid lines), with 95% confidence interval around the predicted mean (shaded). Source data

As chronic HF is associated with low-grade systemic inflammation^20^ and with the microbiome’s role in host immune regulation^21^, we investigated circulating cytokines and their associations with the gut microbiome. Individual cytokine differences between HF and healthy cohorts were modest (Extended Data Fig. 4a and Supplementary Table 5). This probably reflects a relatively well-compensated HF cohort and the prevalence of insulin resistance within the healthy cohort^22^.

**Fig. 3:**
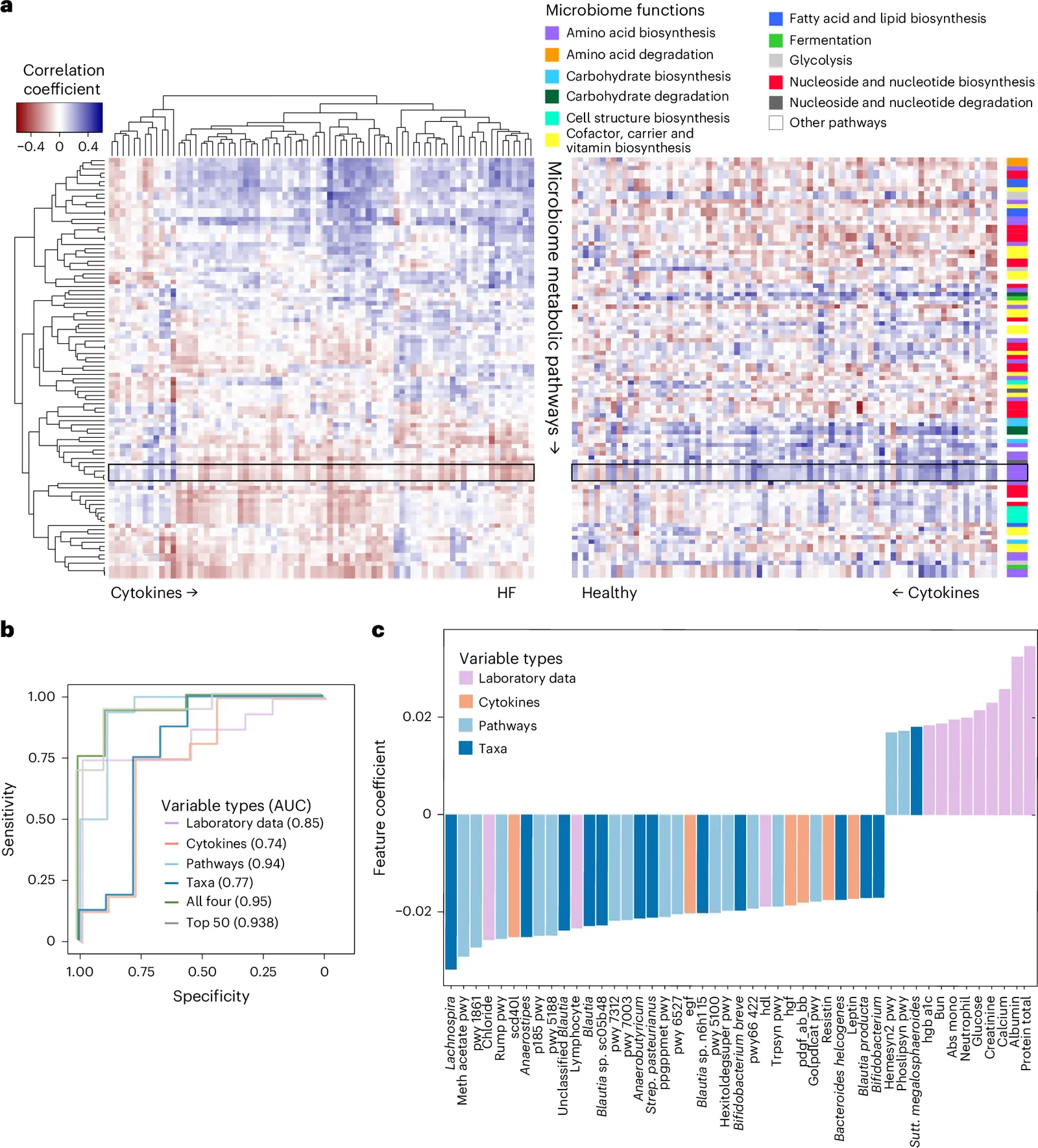
Gut microbiome–host cytokine connections are cohort specific. **a**, Heat map for correlations between the top 100 most abundant microbial pathways (vertical axis) and all 76 cytokines (horizontal axis). Hierarchical clustering on both microbiome and cytokine levels was performed on pairwise Spearman correlations for the baseline samples from the HF cohort (*n* = 58; left) and applied to the pathway–cytokine correlations within the healthy cohort (*n* = 48; right). Black boxes point to the five microbiome pathways with the most divergent correlations between the cohorts, all of which are pathways for arginine and ornithine biosynthesis. **b**, A machine-learning algorithm was used to investigate which data type best classified HF versus healthy participants. The algorithm included clinical laboratory data (53 features), cytokines (76 features), and microbiome taxonomic (all ranks; 553 features) and functional pathway data (306 features) from all visits (*n* = 133). Microbiome pathways resulted in the best classification performance (AUC = 0.94), nearly equivalent to the combined performance of all four data types (AUC = 0.95) and equivalent to the performance of the top 50 individual features (AUC = 0.94). **c**, The top 50 individual features, of which more than half are microbiome related and include genus *Bifidobacterium*. Meth-acetate-pwy, methanogenesis from acetate; pwy 1861, formaldehyde assimilation II (RuMP Cycle); rump-pwy, formaldehyde oxidation I; p185 pwy, formaldehyde assimilation III; pwy 5188, tetrapyrrole biosynthesis I (from glutamate); pwy 7312, dTDP-d-beta-fucofuranose biosynthesis; pwy 7003, glycerol degradation to butanol; ppgppmet pwy, ppGpp biosynthesis; pwy 6527, stachyose degradation; pwy 5100, pyruvate fermentation to acetate and lactate II; hexitoldegsuper pwy, superpathway of hexitol degradation (bacteria); pwy66 422, d-galactose degradation V (Leloir pathway); trpsyn pwy, l-tryptophan biosynthesis; golpdlcat pwy, superpathway of glycerol degradation to 1,3-propanediol; hemesyn2 pwy, heme biosynthesis II (anaerobic); phoslipsyn pwy, superpathway of phospholipid biosynthesis I (bacteria). Source data

Microbiome–cytokine correlation patterns differed substantially between cohorts for both microbial pathways (Fig. 3a) and genera (Extended Data Fig. 4b and Supplementary Table 5). A prominent example involved l-arginine and ornithine biosynthetic pathways (Fig. 3a (black box) and Extended Data Fig. 4c), which are relevant to the regulation of gut permeability and the host inflammatory state^23^, and production of nitric oxide and polyamines, both linked to HF pathophysiology^24,25^. Several of these pathways were depleted in the HF cohort. While the degree to which the microbiome contributes to the host arginine pool is unknown, diminished global arginine bioavailability has been associated with more severe HF^26^. In the HF cohort, the arginine and ornithine biosynthetic potential was inversely correlated with circulating tumor necrosis factor (TNF), with healthy participants having the opposite pattern (Extended Data Fig. 4c). Within the HF cohort, patients with high l-arginine and ornithine pathway abundances also had lower C-reactive protein levels (*P* = 0.001; Extended Data Fig. 4d). Although these correlations were modest, the cohort-specific patterns suggest that microbiome–immune system interactions are systemically reorganized in chronic HF.

### Gut microbial pathways distinguish chronic HF from healthy states

As different molecular types can reflect distinct disease facets, we evaluated how well our multi-omic datasets distinguished chronic HF from healthy states. We used clinical laboratory data (53 features), cytokines (76 features), microbiome taxa (553 features) and pathway data (306 features) to develop machine learning HF classification models (Methods). The full multi-omic model achieved an area under the curve (AUC) of 0.95, while a model based on only microbiome pathways achieved an AUC of 0.94 (Fig. 3b). Similar performance was obtained using the top 50 most important features (AUC = 0.94), of which 32 were microbiome features, including *Bifidobacterium* and *Lachnospira*, and pathways related to methanogenesis and formaldehyde detoxification (Fig. 3c). These findings suggest that gut microbiome features capture substantial information associated with chronic HF.

### Gut microbial taxa and pathways are associated with chronic HF severity and clinical outcomes

**Fig. 4:**
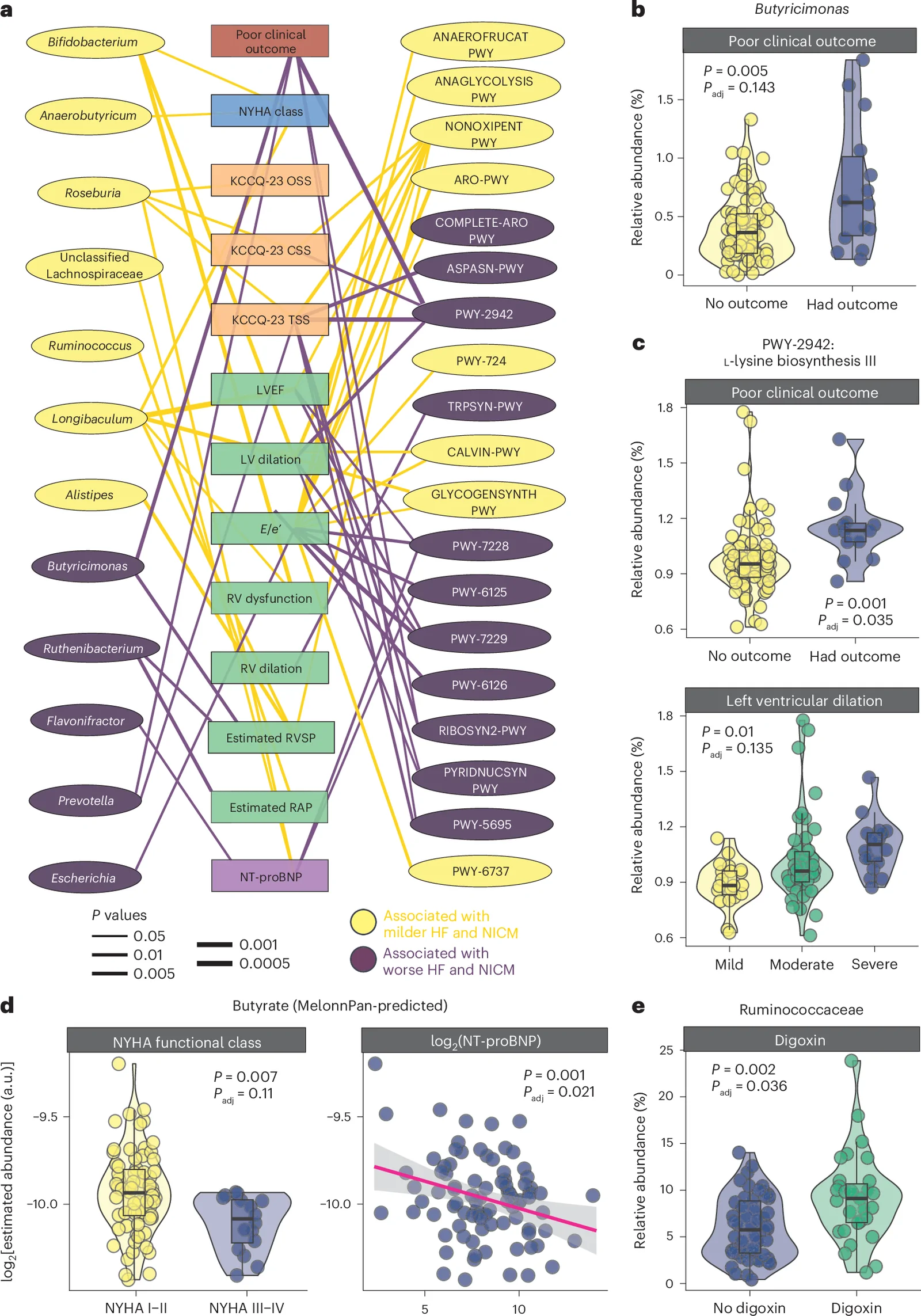
HF and cardiomyopathy severity metrics are associated with levels of gut microbiome taxa, metabolic pathways and predicted gut microbial metabolites. **a**, Gut microbial taxa and pathways are associated with HF severity (NYHA functional class, KCCQ-23 scores, echocardiographic measurements, NT-proBNP and risk of poor clinical outcome (composite of death or hospice, heart transplant or LVAD implant)). **b**,**c**, Genus *Butyricimonas* (**b**) and l-lysine biosynthesis III pathway (PWY-2942) (**c**) were enriched in patients with poor clinical outcome (*n* = 12). PWY-2942 was associated with LV enlargement (*n* = 58) (**c**). **d**, Predicted SCFA butyrate was associated with milder HF (NYHA I–II (*n* = 45), NT-proBNP (*n* = 59)). **e**, Patients prescribed digoxin (*n* = 19) had enrichment of family Ruminococcaceae. In **a**–**e**, linear mixed-effects models (microbiome features as dependent variables, HF severity metric or digoxin as independent variables) were adjusted for age, sex, race (white versus other), BMI and diet (fixed effects), and participant (random effect). The digoxin model additionally accounted for NYHA. Two-sided *t*-tests with Satterthwaite’s degrees of freedom approximation were used. In boxplots (**b**–**e**), the center represents the median, box limits show the IQR and whiskers extend to 1.5 × IQR. The scatter plot (**d**) shows linear fit (pink line), with 95% confidence interval around the predicted mean (shaded). Each panel shows all available patient data, including repeated measures. **a** shows nominally significant associations. The Benjamini–Hochberg method was used to adjust for multiplicity (**b**–**e**). OSS, overall summary score; CSS, clinical summary score; TSS, total symptom score; LVEF, left ventricular ejection fraction; LV, left ventricle; *E*/*e*′, early mitral inflow velocity (*E*)/early diastolic mitral annular velocity (*e*′); RV, right ventricle; RVSP, right ventricular systolic pressure; RAP, right atrial pressure; ANAEROFRUCAT-PWY, homolactic fermentation; ANAGLYCOLYSIS-PWY, glycolysis III (from glucose); NONOXIPENT-PWY, pentose phosphate pathway (non-oxidative branch); ARO-PWY, chorismate biosynthesis I; COMPLETE-ARO-PWY, superpathway of aromatic amino acid biosynthesis; ASPASN-PWY, superpathway of l-aspartate and l-asparagine biosynthesis; PWY-2942, l-lysine biosynthesis III; PWY-724, superpathway of l-lysine, l-threonine and l-methionine biosynthesis II; TRPSYN-PWY, l-tryptophan biosynthesis; CALVIN-PWY, Calvin-Benson-Bassham cycle; GLYCOGENSYNTH-PWY, glycogen biosynthesis I (from ADP-D-Glucose); PWY-7228, superpathway of guanosine nucleotides de novo biosynthesis I; PWY-6125, superpathway of guanosine nucleotides de novo biosynthesis II; PWY-7229, superpathway of adenosine nucleotides de novo biosynthesis I; PWY-6126, superpathway of adenosine nucleotides de novo biosynthesis II; RIBOSYN2-PWY, flavin biosynthesis I (bacteria and plants); PYRIDNUCSYN-PWY, NAD biosynthesis I (from aspartate); PWY-5695, urate biosynthesis/inosine 5′-phosphate degradation; PWY-6737, starch degradation V. Source data

Given the involvement of gut microbial metabolites in HF pathophysiology^9,10,15^, we tested associations between gut microbial features and HF severity, including physician-adjudicated NYHA functional class, patient-reported KCCQ-23 scores, echocardiographic parameters, circulating N-terminal pro-B-type natriuretic peptide (NT-proBNP) and poor clinical outcome. After adjustment for demographics, BMI and diet, several microbial genera and pathways were associated with individual HF severity metrics (Fig. 4a). *Butyricimonas* was associated with poor clinical outcome (*P* = 0.005, *P*_adj_ = 0.143; Fig. 4b) and worse estimated RV systolic pressure (RVSP; *P* = 0.011, *P*_adj_ = 0.116). *Bifidobacterium* was associated with milder cardiomyopathy, including less LV enlargement (*P* = 0.005, *P*_adj_ = 0.066) and less RV dysfunction (*P* = 0.003, *P*_adj_ = 0.083), and, although only nominally, inversely associated with NYHA class (*P* = 0.042) (Supplementary Table 6).

**Extended Data Fig. 5:**
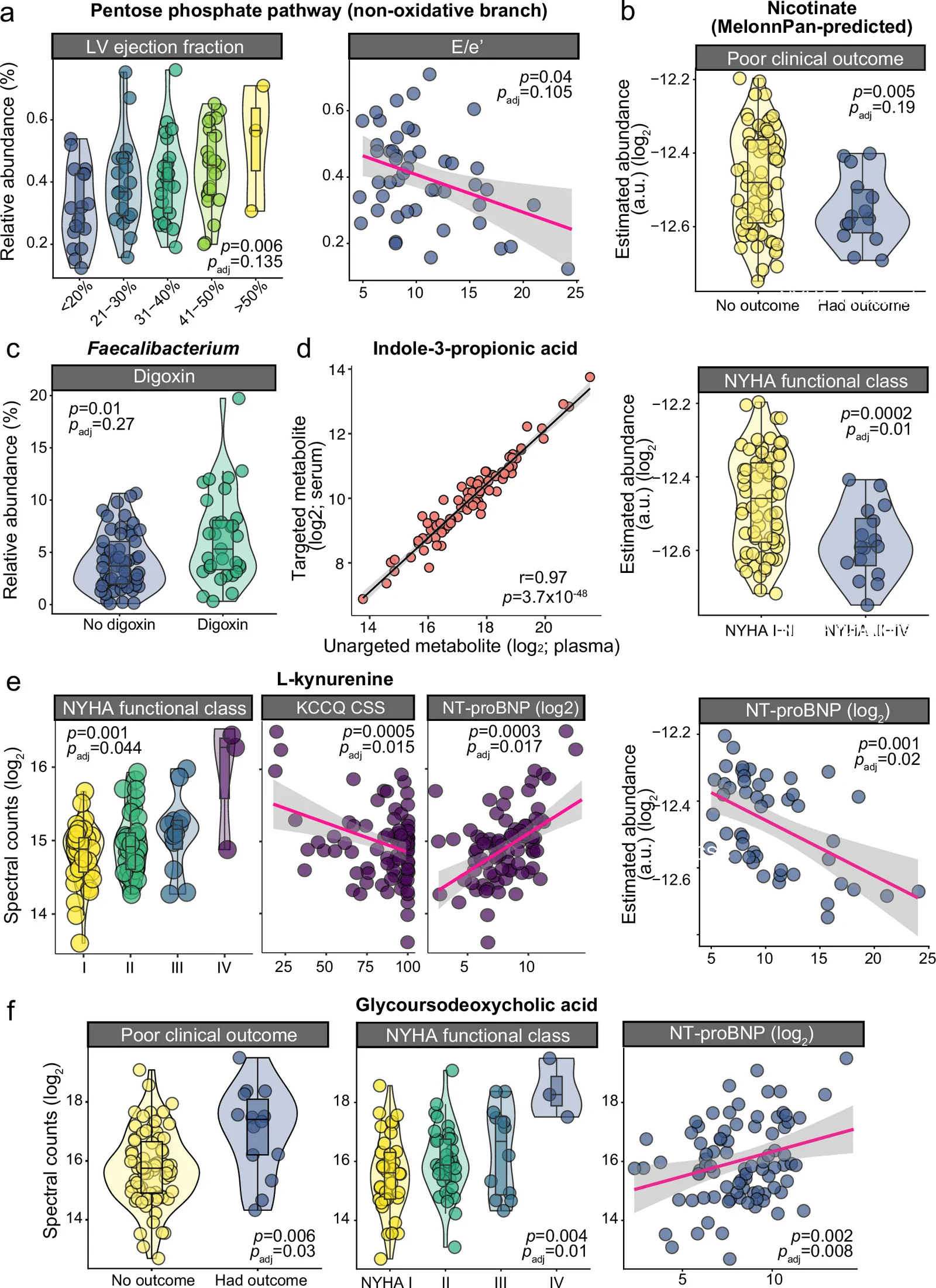
Gut microbiome functional community features and metabolites were associated with HF and cardiomyopathy severity. **a**, In this HF cohort (*n* = 59, with total of 86 samples), pentose phosphate pathway (non-oxidative branch) was associated with better left ventricular (LV) function (systolic and diastolic). **b**, Predicted butyrate levels were associated with milder HF. **c**, Patients prescribed digoxin (*n* = 19) had higher relative abundance of genus *Faecalibacterium*, though this was only nominally significant. **d**, Circulating indole-3-propionic acid (IPA) assessed via untargeted and targeted metabolomics (plasma and serum samples, respectively) were highly correlated. **e**, Circulating L-kynurenine was associated with more severe HF. **f**, Glycoursodeoxycholic acid, a secondary conjugated bile acid, was associated with more severe HF. In **a-c**, **e-f**, linear mixed-effects models (HF severity metric as independent variables, microbiome and circulating features as dependent variables) were adjusted for age, sex, race (white versus other), body mass index, and diet (fixed effects), and participant (random effect). Two-sided *t*-tests with Satterthwaite’s degrees of freedom approximation were used. Digoxin model (**c**) additionally accounted for New York Heart Association class. Benjamini-Hochberg method adjusted for multiplicity. In **d**, Pearson correlation was used for IPA comparison, and *p*-value calculated from a two-tailed Student’s *t*-test. In boxplots, the center is median, box limits show interquartile range (IQR), whiskers extend to 1.5*IQR. The scatter plots (**a-b, e-f**) show linear fit (pink line), with 95% confidence interval around the predicted mean (shaded). Each panel shows all available patient data, including repeated measures. E/e’, early mitral inflow velocity (E)/early diastolic mitral annular velocity (e’); NT-proBNP, N-terminal pro-B-type natriuretic peptide; KCCQ, Kansas City Cardiomyopathy Questionnaire; CSS, clinical summary score. Source data

Microbiome functional analysis identified PWY-2942: l-lysine biosynthesis III pathway as associated with more severe HF, including poor clinical outcome (*P* = 0.001, *P*_adj_ = 0.035; Fig. 4a,c), more LV enlargement (*P* = 0.01, *P*_adj_ = 0.135; Fig. 4a,c) and KCCQ total symptom score (TSS; *P* = 0.002, *P*_adj_ = 0.063; Fig. 4a). Pathways for de novo adenosine and guanosine biosynthesis were also associated with more severe HF, while the non-oxidative pentose phosphate pathway was associated with milder HF (Fig. 4a and Extended Data Fig. 5a). Predicted gut microbial production of SCFA butyrate was associated with milder HF (Fig. 4d). Altogether, these findings identify taxonomic and functional microbiome signatures associated with HF severity, although it remains to be determined whether these contribute to disease progression or reflect its physiological consequences.

### Digoxin is associated with altered gut microbiome in chronic HF

As medications can influence microbiome composition^27^, we assessed associations between microbiome and commonly prescribed HF medications. We focused on mineralocorticoid receptor antagonists (MRAs), diuretics and digoxin, prescribed to 81%, 52% and 32% of patients, respectively. Diuretic or MRA use was not associated with microbiome features (Supplementary Table 7). Digoxin was associated with the family Ruminococcaceae (*P* = 0.002, *P*_adj_ = 0.036; Fig. 4e), an association probably driven by genus *Faecalibacterium* and species *Faecalibacterium prausnitzii*, although these associations were not significant after multiplicity correction (Extended Data Fig. 5c). These findings suggest a previously unrecognized association between digoxin and the beneficial commensal *F. prausnitzii*^28,29^, previously reported as depleted in HF^4,30^, cardiometabolic diseases and aging^30,31^. Thus, we suggest a possible mechanism through which digoxin may influence microbiome composition, potentially contributing to its clinical benefits in chronic systolic HF^32,33^.

### Gut microbiome metabolites are associated with chronic HF severity

**Fig. 5:**
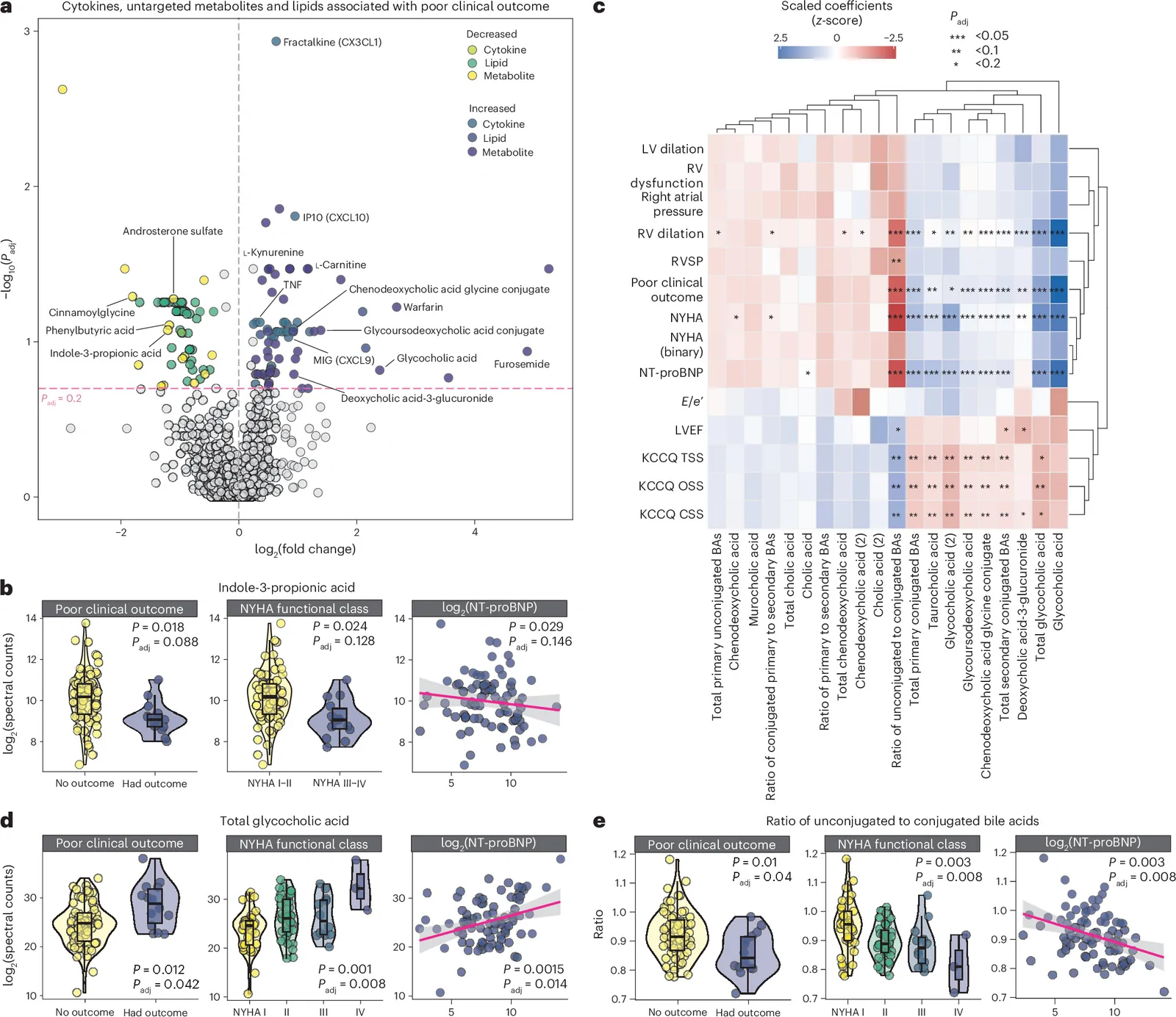
Chronic HF severity is associated with broad patterns of host immune and metabolic dysregulation, including many microbiome-related metabolites. **a**, Circulating cytokines, untargeted metabolites and lipids are associated with poor clinical outcome (composite of death or hospice, heart transplant or LVAD implant). log_2_(fold change) was calculated for feature mean values for each group. **b**, IPA (targeted metabolomics) was associated with lower risk of poor clinical outcome (*n* = 47) and overall milder HF (NYHA I–II *n* = 45, NT-proBNP *n* = 59). **c**, BAs showed broad and strong associations with metrics indicative of more severe HF. Hierarchical clustering used coefficients derived from multivariate linear mixed models, median normalized and scaled by *z*-score. **d**,**e**,Total circulating glycocholic acid (primary conjugated BA) was associated with more severe HF (poor clinical outcome *n* = 12, NYHA III–IV *n* = 14, NT-proBNP *n* = 59) (**d**), whereas the total unconjugated-to-conjugated BA ratio was associated with milder HF (no poor clinical outcome *n* = 47, NYHA I–II *n* = 45, NT-proBNP *n* = 59) (**e**). In **a** and **b**–**e**, linear mixed-effects models (circulating features as dependent variables, HF severity as independent variable) were adjusted for age, sex, race (white versus other), BMI and diet (not included in the cytokine model) (fixed effects), and participant (random effect). Two-sided *t*-tests with Satterthwaite’s degrees of freedom approximation were used. In boxplots (**b**, **d** and **e**), the center is the median, box limits show the IQR and whiskers extend to 1.5 × IQR. The scatter plots (**b**, **d** and **e**) show linear fit (pink line), with a 95% confidence interval around the predicted mean (shaded). Each panel shows all available patient data, including repeated measures. The Benjamini–Hochberg method was used to adjust for multiplicity (**a**–**e**). Source data

Chronic HF is a state of immune, neurohormonal and metabolic dysregulation^20,34^. Thus, we examined associations between circulating cytokines, metabolites and lipids with HF severity. Of 76 assayed cytokines, 36 were associated with at least 1 HF severity metric (Fig. 5a and Supplementary Table 8).

Circulating SCFAs were not associated with HF severity. By contrast, higher circulating IPA was associated with milder HF, including lower risk of poor clinical outcome, lower NYHA class, *E*/*e*′, RVSP and NT-proBNP, and higher KCCQ scores (Fig. 5b and Supplementary Table 8). IPA measurements from targeted and untargeted metabolomics were strongly correlated (Extended Data Fig. 5d). Data validity is further corroborated by lower levels of anabolic hormones (for example, androsterone sulfate, dehydroisoandrosterone sulfate) in patients with poor clinical outcome from advanced HF, a known catabolic state^35^. In addition, detected metabolites of cardiac medications were associated with disease severity in predictable patterns (Fig. 5a). Untargeted metabolomics identified multiple other microbiome-derived circulating metabolites associated with HF severity (Fig. 5a and Supplementary Table 8), including l-kynurenine (Extended Data Fig. 5e), another tryptophan metabolite, which was associated with more severe HF, consistent with previous studies^36^.

Circulating bile acids (BAs) showed broad associations with HF severity (Fig. 5a,c–e and Supplementary Table 9). These cardio- and metabolically active molecules are regulated by gut microbial metabolism, whereby gut microorganisms deconjugate primary and secondary conjugated BAs during their enterohepatic recirculation. Several conjugated BAs (for example, glycocholic acid; Fig. 5a,c,d) were associated with more severe HF, while a higher ratio of unconjugated-to-conjugated BAs was associated with milder HF (Fig. 5c,e), corroborating chronic HF-related BA dysregulation^37^, probably related to gut microbiome shifts. Collectively, our results support widespread associations between HF severity and circulating metabolites, many derived from or influenced by microbiome metabolism, implicating their role in HF pathophysiology.

### *Bifidobacterium* abundance is associated with HF improvement over time

**Extended Data Fig. 6:**
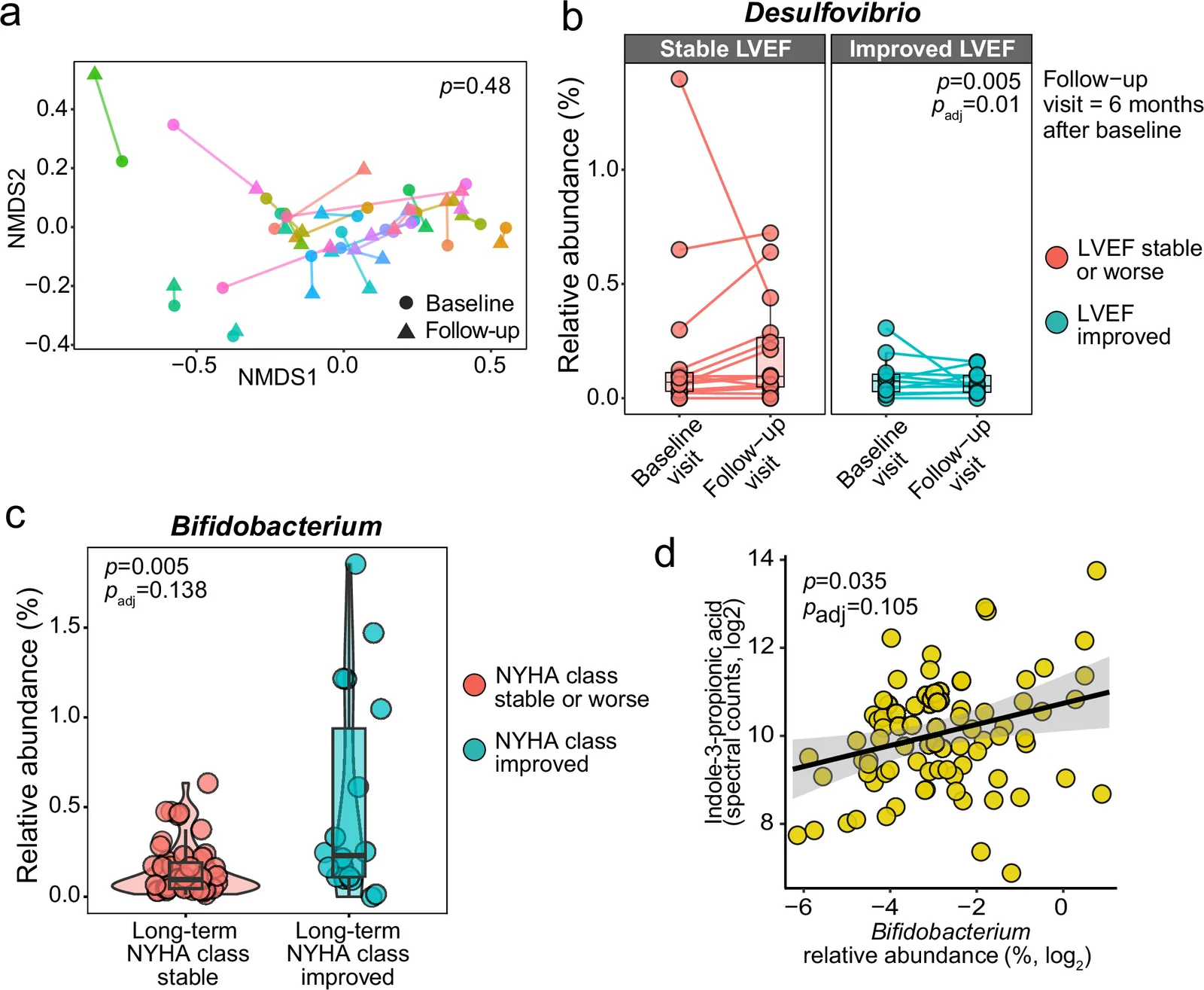
*Bifidobacterium* relative abundance was associated with HF long-term clinical improvement. **a**, Comparing gut microbiome of patients with follow-up sampling (*n* = 26, total of 52 samples), beta-diversity was not significantly different between baseline and follow-up visits (PERMANOVA *p* > 0.05; non-metric multidimensional scaling ordination of microbiome pathways), with samples from the same patient (different colors) clustering together instead. **b**, *Desulfovibrio* was negatively associated with left ventricle ejection fraction (LVEF) improvement over time (*n* = 11). **c**, *Bifidobacterium* was associated with long-term New York Heart Association (NYHA) functional class improvement from baseline (*n* = 7 of 51 patients with long-term clinical follow-up data available, obtained through the electronic medical record). **d**, *Bifidobacterium* relative abundance (log_2_-transformed for visualization) was associated with circulating indole-3-propionic acid (IPA) (targeted metabolomics) (*n* = 58, with 87 samples total). In **b-c**, linear mixed-effect models (HF clinical change as independent variable, gut microbiome feature as dependent) accounted for age, sex, race (white versus other), body mass index, and dietary patterns (fixed effects), and participant as random effect. In **d**, *Bifidobacterium* was modeled as independent, and circulating IPA as the dependent variable (all data shown). In boxplots, the center is median, box limits show interquartile range (IQR), whiskers extend to 1.5*IQR. The scatter plot shows linear fit (line), with 95% confidence interval around the predicted mean (shaded). Benjamini-Hochberg method was used to adjust for multiplicity (**b**-**d**). PERMANOVA, permutational multivariate analysis of variance. Source data

We investigated longitudinal gut microbiome and multi-omic changes in 26 participants in our HF cohort. Microbiome beta-diversity did not differ substantially between visits (Extended Data Fig. 6a and Supplementary Table 10), with samples clustering primarily by participant. No individual microbial or host multi-omic features changed significantly over time after multiple comparison correction (Supplementary Table 10).

**Fig. 6:**
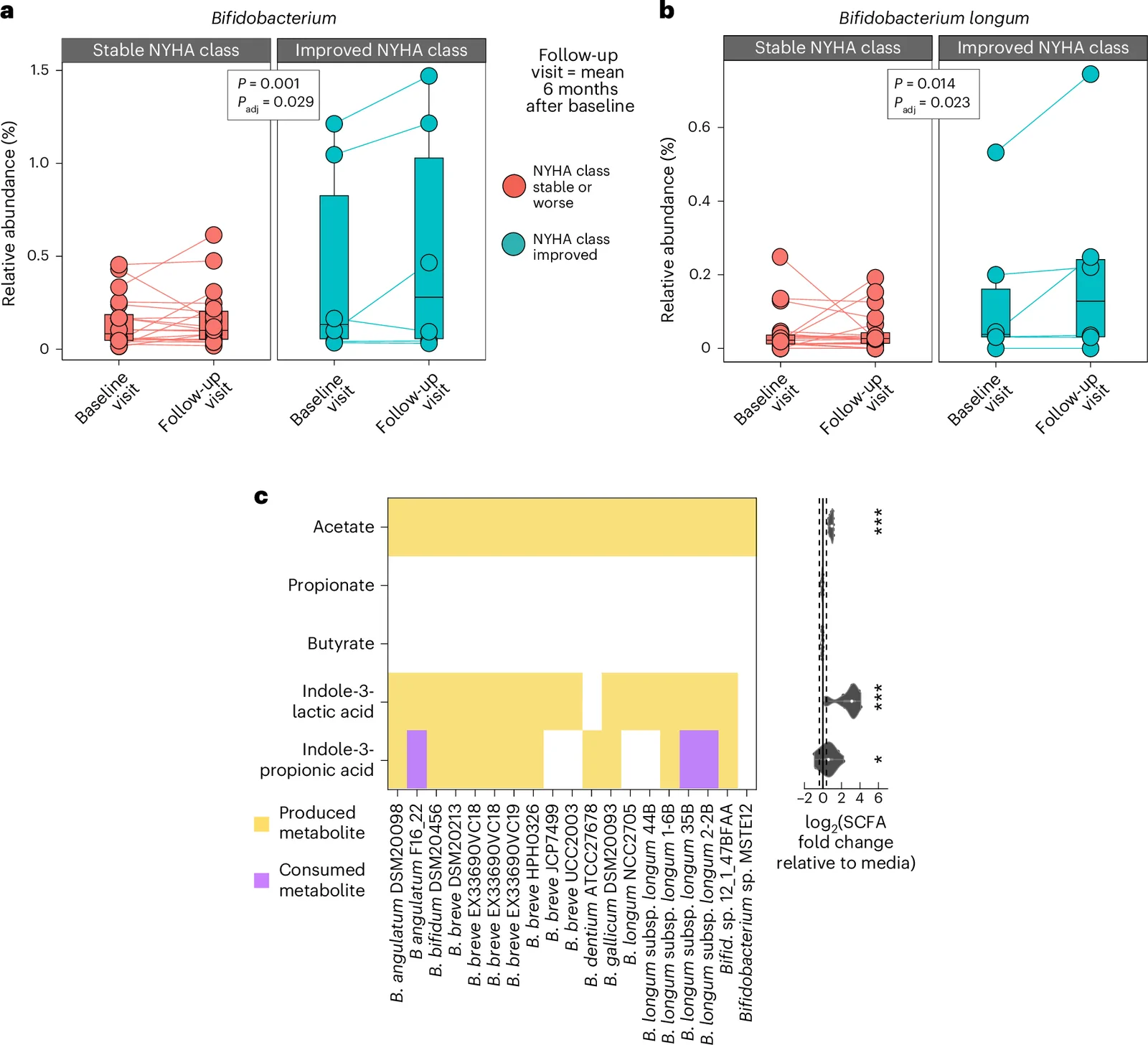
*Bifidobacterium* relative abundance is associated with symptomatic HF improvement over time. **a**,**b**, The gut microbiome genus *Bifidobacterium* (**a**) and its most abundant species *B. longum* (**b**) were more abundant at baseline and increased over time in individuals whose HF functional status improved over time (*n* = 6, defined as an improvement in at least one NYHA functional class between baseline and follow-up visits), compared with individuals who remained stable or worsened over time (*n* = 20). **c**, Left: targeted exometabolomics data for various *Bifidobacterium* strains in vitro. For each metabolite, fold changes were calculated relative to the base medium (Mega Media). Only changes >30% are plotted. Right: quantification of metabolite fold change for 19 species. The dashed vertical lines represent 1 s.d. of the log-fold change calculated from media control replicates. ***P_adj_ < 0.001; *P_adj_ < 0.05. Two-tailed Student’s *t*-tests. Linear mixed models with age, sex, race (white versus other), BMI and dietary patterns as fixed effects and participant as a random effect were used in **a** and **b**, followed by Benjamini–Hochberg adjustment. In **a** and **b**, categorical NYHA improvement was modeled as the independent variable, with relative abundance of the genus *Bifidobacterium* and species *B. longum*, respectively, as the dependent variable. In boxplots, the center line represents the median, box limits show the IQR and whiskers extend to 1.5 × IQR. *B. angulatum*, *Bifidobacterium angulatum*; *B. bifidum*, *Bifidobacterium bifidum*; *B. gallicum*, *Bifidobacterium gallicum*; *Bifid.* sp., *Bifidobacterium* species. Source data

We next tested whether microbiome features were associated with divergent clinical trajectories. Between visits, 6, 11 and 8 patients experienced improvement in NYHA class, LVEF or KCCQ-23 overall summary score (OSS), respectively. *Bifidobacterium* abundance was associated with improved NYHA class (*P* = 0.001, *P*_adj_ = 0.029; Fig. 6a), while *Desulfovibrio* was inversely associated with LVEF improvement (*P* = 0.005, *P*_adj_ = 0.01; Extended Data Fig. 6b). No microbiome features were significantly associated with improved KCCQ-23 OSS (Supplementary Table 11). Targeted metabolomics-assayed IPA was associated with NYHA class improvement (*P* = 0.036, *P*_adj_ = 0.18; Supplementary Table 11). Analyses of other multi-omic analytes (cytokines, untargeted metabolites, lipids) revealed potential associations with HF improvement (including two conjugated BAs inversely associated with NYHA class improvement), although none remained significant after multiple comparison correction. Among the 51 patients with long-term clinical follow-up, *Bifidobacterium* abundance was associated with NYHA class improvement (*P* = 0.005, *P*_adj_ = 0.138; Extended Data Fig. 6c). Long-term LVEF improvement (*n* = 10) was not associated with microbiome signatures (Supplementary Table 11).

Having identified genus *Bifidobacterium* as associated with HF improvement, we queried the contribution of its individual species. *Bifidobacterium longum* (Fig. 6b), *Bifidobacterium* *breve* and *Bifidobacterium pseudocatenulatum* were associated with between-visit NYHA class improvement (*P* = 0.013, *P* = 0.014 and *P* = 0.018, respectively; *P*_adj_ = 0.024 for all; Supplementary Table 12), as well as with several HF severity metrics indicating milder HF. Our findings identify *Bifidobacterium* abundance as a candidate biomarker of clinical HF improvement that warrants validation in larger longitudinal cohorts.

### *Bifidobacterium* species produce IPA in vitro

To explore potential mechanisms linking *Bifidobacterium* to HF progression, we tested associations between *Bifidobacterium* relative abundance and circulating metabolites (Supplementary Table 13). *Bifidobacterium* was associated with circulating IPA (assayed by targeted metabolomics) (*P* = 0.035, *P*_adj_ = 0.105; Extended Data Fig. 6d), although the relationship was sensitive to outliers. No statistically significant associations were identified with individual BAs even though *Bifidobacterium* species are known to express BA-deconjugating bile salt hydrolases (Supplementary Table 13).

We tested whether *Bifidobacterium* can produce SCFAs and indole metabolites in vitro. Consistent with a previous study^38^, we found that most of the 19 tested *Bifidobacterium* strains produced and secreted acetate and ILA. A smaller subset of *Bifidobacterium* strains also produced IPA (Fig. 6c and Supplementary Table 14). These findings suggest that *Bifidobacterium* species can produce cardioprotective metabolites such as IPA and support a plausible metabolic link between *Bifidobacterium* and HF physiology.

## Discussion

**Fig. 7:**
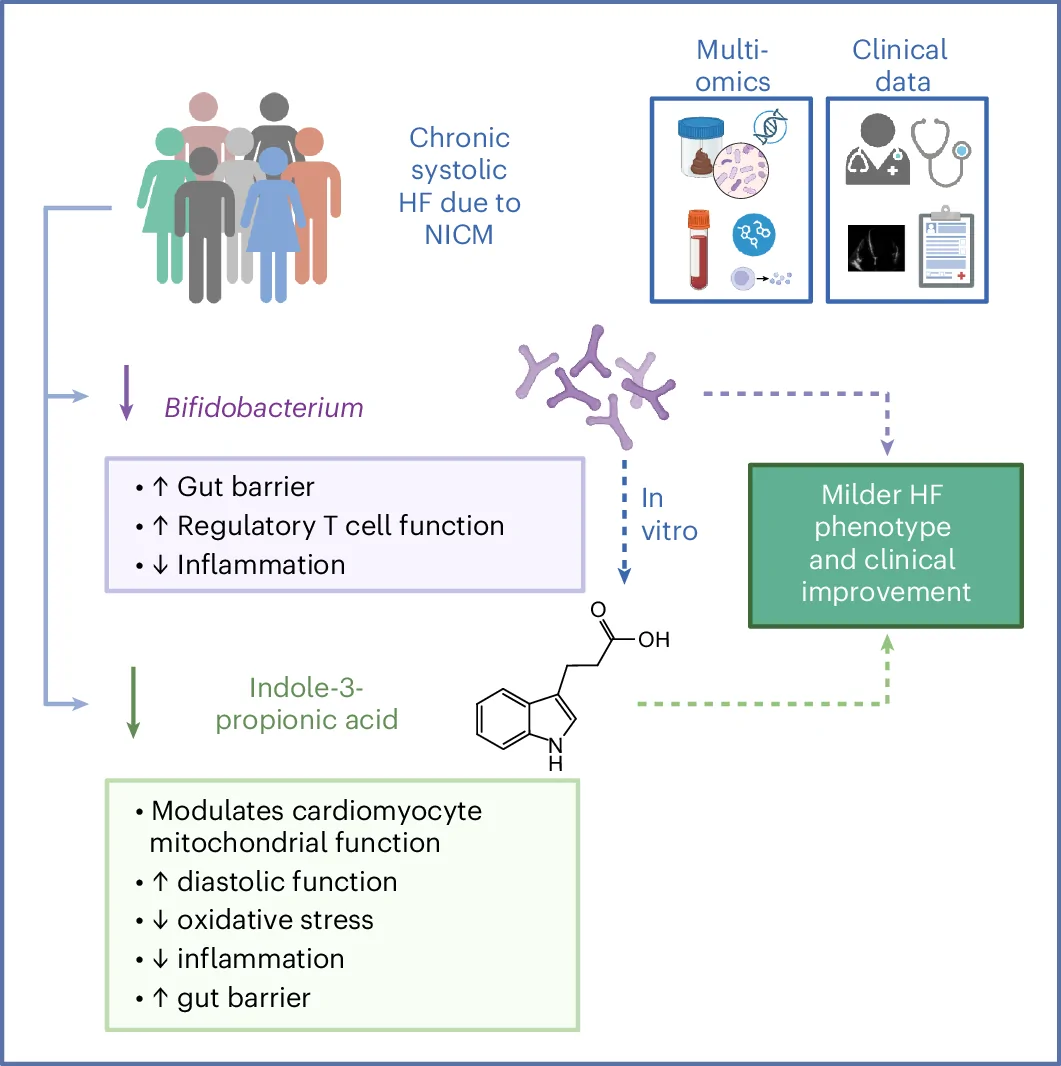
Summary of study findings. In adults with NICM-related chronic systolic HF, compared with healthy controls, the relative abundance of *Bifidobacterium* is reduced, as is the circulating level of gut microbial metabolite IPA. This cardioprotective metabolite, previously implicated in biological processes dysregulated in chronic HF, is produced by *Bifidobacterium* species in vitro. Both *Bifidobacterium* and IPA are associated with milder HF phenotype and HF improvement over time (expressed as between-visit change in the NYHA functional class). Figure created in BioRender; Mamic, P. https://biorender.com/kfsgybk (2026).

Our multi-omic study of patients with NICM-related systolic HF, with longitudinal profiling and clinical follow-up for a subset of the cohort, identified substantial differences in gut microbiome taxonomic and functional composition and differential microbiome–immune system connections compared with healthy participants. Furthermore, we uncovered gut microbiome signatures of chronic systolic HF severity and identified *Bifidobacterium* as a candidate biomarker of HF improvement (Fig. 7).

These findings align with previous studies showing gut microbiome alterations in HF^4–6,8^, including reduced alpha-diversity and depletion of SCFA-producing microorganisms that may influence systemic inflammatory tone, and could potentially be modulated by diet. Evidence on *Bifidobacterium* is mixed: two small studies found enrichment in patients with decompensated^39^ and severe HF^40^, while larger studies support our finding of depletion^5,8^. We additionally observed enrichment of pro-inflammatory taxa and potential pathogens in HF, which may contribute to gut barrier dysfunction and disease progression^4,12^.

Loss of SCFA fermentative pathways and predicted butyrate production in our metagenomic analysis corroborate the taxonomic findings. SCFAs regulate blood pressure, cardiac hypertrophy, fibrosis and repair^9,10,15^. Despite differences in their biosynthetic potential between cohorts, systemic SCFA levels were similar, possibly reflecting differential SCFA gut epithelial or hepatic utilization. The HF-associated gut microbiome also showed enriched fatty acid beta-oxidation, suggesting an oxygen-rich environment favoring facultative anaerobes, many pro-inflammatory or pathogenic^14,15^. Reduced formaldehyde detoxification alongside enriched folate biosynthesis may increase luminal formaldehyde levels, potentially promoting epithelial oxidative stress and inflammation^41^.

Our multi-omic machine learning model combining clinical, cytokine and microbiome data accurately distinguished chronic HF from the healthy state. Models using only microbiome pathways or the top 50 features performed similarly, with microbiome features predominating, including SCFA biosynthesis and formaldehyde detoxification pathways, and genera *Bifidobacterium* and *Lachnospira*. Our findings suggest that microbiome features, particularly functional ones, may potentially serve as HF diagnostic biomarkers, although validation in larger cohorts with varied disease severity and against other chronic inflammatory conditions is needed. The microbiome prominence in this multi-omic HF classification model suggests its important role in HF physiology and potential mechanistic involvement.

Our study also identified gut microbiome signatures of chronic NICM-associated HF severity. *Butyricimonas* was associated with poor clinical outcome. While identified as a butyrate producer and associated with insulin sensitivity^42^, *Butyricimonas* was also enriched in patients with coronary artery disease^43^. Microbiome l-lysine biosynthesis was enriched in the HF cohort and associated with more severe HF, consistent with l-lysine metabolism dysregulation in pressure-overload HF models^44^. Predicted gut butyrate levels were inversely correlated with HF severity, reinforcing the SCFA relevance in HF physiology, despite lacking associations between circulating SCFAs and HF severity metrics.

*Bifidobacterium* abundance was associated with milder cardiomyopathy and functional improvement, a finding corroborated by another study^45^. Our multi-omic exploration of potential HF-relevant *Bifidobacterium* mechanisms prioritized circulating IPA. This microbiome-derived metabolite of dietary tryptophan was associated with multiple metrics of milder HF and disease improvement in our cohort. IPA exerts direct and indirect cardioprotective effects: it modulates cardiomyocyte mitochondria^17^ and mitigates anthracycline^18^ and immune checkpoint inhibitor cardiotoxicity^19^. IPA also reduces oxidative stress and inflammation, and improves gut barrier function, alleviating diastolic dysfunction in mice^16^. Circulating IPA is reduced in patients with diastolic HF^16^, compared with healthy controls. Our report of IPA reduction in the chronic systolic HF cohort is consistent with a recent report of IPA as a biomarker in both compensated and decompensated systolic HF^46^. Supporting this mechanistic link, we showed that multiple *Bifidobacterium* species found in our cohort produced IPA in vitro. However, circulating IPA in vivo reflects integrated community metabolism, dietary substrate availability and host handling distributed across multiple taxa; accordingly, *Bifidobacterium* abundance may not reliably track circulating IPA levels in a heterogeneous clinical cohort. Nonetheless, *Bifidobacterium* probiotic interventions increase circulating IPA in experimental models^47^ and in humans^48^, supporting plausible modulation of host IPA exposure directly and via community-level interactions.

Beyond tryptophan metabolism, *Bifidobacterium* may influence HF through BA metabolism. We observed extensive BA dysregulation in our HF cohort, with conjugated BA accumulation in patients with more severe disease. Although *Bifidobacterium* was not associated with individual circulating BAs in our linear multivariate analyses, it may still contribute to microbiome-wide bile salt hydrolase activity that shapes the BA pool.

Gut microbiome-linked HF improvements probably involve multiple complementary mechanisms across distinct taxa and metabolites acting on diverse host targets. Elucidating these interactions may uncover therapeutic opportunities. Future clinical studies should evaluate whether *Bifidobacterium* probiotic supplementation influences HF physiology and outcomes, and whether proposed mechanisms (for example, IPA production, BA metabolism modulation) mediate these effects.

### Strengths

This study integrates host multi-omics, metagenomic sequencing (enabling both taxonomic and functional microbiome assessment), detailed clinical data and longitudinal sampling for a subset of participants. This approach enabled deep dissection of chronic HF-associated gut microbiome composition and its links with host immunity and metabolism, identification of microbiome signatures associated with HF severity and trajectories, and proposal of mechanisms potentially implicated in HF physiology. Dietary data collection enabled correction for this important factor.

### Limitations

Our single-center study has demographic limitations, with predominantly white and, in the HF cohort, male participants. Technical differences between cohorts limited certain comparisons: untargeted metabolomic and targeted lipidomic data were not directly comparable, and stool collection methods differed. While validation suggested minimal collection method impact, residual confounding cannot be excluded. Disentangling disease-associated microbiome changes from medication effects in heavily medicated patients is inherently limited. We assessed only select HF medications, identifying associations between digoxin and Ruminococcaceae, but broader medication effects remain incompletely resolved. Dedicated studies will need to define the impact of individual medications on HF-associated microbiomes. Finally, results may not generalize to all patients with HF, as our study included only patients with NICM-related systolic HF, excluding patients with treated diabetes and other serious comorbidities. Our relatively small, well-compensated cohort probably additionally skewed results. Even so, we detected microbiome and multi-omic signals associated with HF severity and clinical change, although validation in larger cohorts is needed.

### Summary

In this multi-omic study of NICM-related chronic HF, with longitudinal sampling and clinical follow-up in a subset of patients, we define HF-associated taxonomic and functional gut microbiome signatures and identify microbiome features linked to HF severity. *Bifidobacterium*, depleted in chronic HF, is directly associated with clinical improvement over time. We identify circulating IPA, a gut microbiome metabolite with cardioprotective properties, as associated with milder HF phenotype and clinical improvement. Multiple *Bifidobacterium* species produce IPA in vitro, suggesting a plausible mechanism through which *Bifidobacterium* may impact HF physiology and disease progression. Future clinical studies of *Bifidobacterium* probiotic supplementation should be pursued to validate *Bifidobacterium* as a biomarker of HF improvement and evaluate its potential role as an active contributor to HF physiology.

## Methods

### Study population

#### Patients with chronic HF

Between July 2018 and March 2020, 59 adults from Stanford Cardiomyopathy Clinic were enrolled in the study^49^. Participants provided written informed consent under Stanford University Institutional Review Board research protocol 44967. We included adults (ages 18–75) with established NICM and systolic HF. Exclusion criteria (Supplementary Table 1) included primary ischemic cardiomyopathy, complex congenital heart disease, treated diabetes, advanced chronic kidney disease (stage 4–5), advanced liver disease, systemic autoimmune disorder, active malignancy, substantial gastrointestinal disease or history of bowel resection, or appendectomy or cholecystectomy within 6 months, or conditions requiring systemic immunosuppression. We excluded individuals receiving systemic antibiotics or probiotics within 1 month, or chemotherapy or radiation within 12 months. There was no compensation for study participation.

#### Healthy participants

Healthy controls were recruited from the integrated Personal Omics Profiling (iPOP) study^22,50,51^. This multi-omic, longitudinal study of 109 participants (recruited in 2010–2018) investigated the transition from health to (pre)diabetes. We excluded participants with diabetes, cardiovascular or non-cardiac comorbidities outlined above, or recent antibiotic or probiotic use. A total of 50 iPOP participants were included, each with a single visit.

Validation analysis, done to exclude the possibility of meaningful confounding by sex, included a larger iPOP subset (*n* = 61) with available metagenomic data (Supplementary Information, Extended Data Fig. 2 and Supplementary Table 3).

### Study procedures

#### Stool samples

Patients with HF collected stool samples using OMNIgene-GUT collection kits (OMR-200), which were kept at room temperature for ≤15 days, then aliquoted and frozen at −80 °C; the stabilizing buffer maintains microbiome profiles for ≤60 days (https://www.dnagenotek.com/row/pdf/PD-WP-00056.pdf). Healthy participants previously collected stool per protocol (https://www.hmpdacc.org) without a stabilization buffer, which was immediately frozen at −20 °C, transported on ice and then stored at −80 °C.

#### Blood samples

After overnight fasting (10–12 h), blood was drawn for clinical laboratory tests (Supplementary Table 1) and research assays (cytokines, untargeted and targeted metabolomics, targeted lipidomics). Samples were processed per published protocol^22^ and stored at −80 °C. Clinical laboratory tests were performed in the Stanford Clinical Laboratory per standard protocols.

#### KCCQ-23

Patients with chronic HF completed the KCCQ-23 survey, a validated patient-reported outcome instrument^52^. The 23-question survey generates scores (0–100), whereby lower scores indicate increased symptoms or functional limitations.

#### Dietary questionnaire

Both cohorts completed a food frequency questionnaire (FFQ) modified from the Scottish Collaborative Group FFQ (https://www.foodfrequency.org/), validated in UK adults^53^ and used in multiple US studies^22,51^. This questionnaire assessed habitual consumption of 22 dietary items. FFQ values were converted to numerical values (for example, consuming a food type once per day = 1, 3 or 4 times per week = 0.57 and so on). Missing data (6 out of 136 visits) were imputed using the *k*-nearest neighbors function (‘impute.knn’) in the R ‘impute’ package (v. 1.46.0).

#### Clinical data collection

Demographics, cardiac status, imaging, outcomes (death, hospice, or needing a heart transplant or durable LVAD), comorbidities and medications were extracted from the electronic medical record (EMR).

### Gut microbiome profiling

#### Metagenomic sequencing

##### Library preparation and sequencing

Genomic DNA was extracted using the QIAamp PowerFecal DNA Kit (Qiagen, catalog number 12830-50). Libraries were prepared using the TruSeq DNA PCR-Free Low Throughput Library Prep kit (SKU 20015962) with 1 μg of input DNA. Paired-end sequencing was performed on HiSeqX at Psomagen, generating 3,930,771,952 paired-end 150-bp reads (average 17,548,089 reads per sample). Samples were sequenced in 2 batches; no clear batch effect was observed. The sequencing run with most reads per sample was used for analysis.

##### Metagenome quality control and deduplication

Raw reads were demultiplexed. Adapters and low-quality bases were trimmed using Trimmomatic^54^ (options ‘ILLUMINACLIP:TruSeq3-SE.fa:2:30:10 SLIDINGWINDOW:4:20 MINLEN:50’). Human reads were removed using KneadData (https://github.com/biobakery/kneaddata) against the hg37 reference database. Exact duplicates were removed with clumpify (BBtools) (https://sourceforge.net/projects/bbmap/).

##### Estimating abundances

Bracken v. 2.6 (ref. ^55^; options ‘-r 150 -l S -t 0’) was used to estimate taxonomic abundances of reads classified by Kraken v. 2.0 (ref. ^56^; database v. 2.0.9). Gene families and metabolic pathways were profiled using HUMAnN v. 3.0 (ref. ^57^) using --metaphlan-options="--nproc 16 --unknown_estimation --bowtie2db metaphlan_db_v30 --index mpa_v30_CHOCOPhlAn_201901". Microbiome features with per-sample relative abundance <0.01% (taxa) and <0.001% (pathways) were set to 0%. Features present in <50% of samples were filtered out.

For HF-focused analyses, features were further filtered to those differentially abundant between cohorts or highly abundant in HF, yielding 17 families, 26 genera, 36 species and 31 pathways.

##### Functional enrichment analysis

HUMAnN-identified pathways were hierarchically classified using MetaCyc^58^. Gene Set Enrichment Analysis (GSEA)^59^ identified differentially abundant pathway groups between cohorts.

##### Predicting metabolite from metagenomic data

MelonnPan (v0.99.0)^60^ predicted microbial metabolite levels from gene family estimates, predicting 80 of 107 metabolites from the original MelonnPan validation dataset. Predicted metabolites were log_2_ transformed before downstream analyses.

### Host circulating multi-omic profiling

#### Targeted quantification of gut microbial metabolites

##### Sample preparation

Samples were processed as previously described^61^. Bacterial supernatants were diluted 1:100 into extraction buffer (90% HPLC-grade water (Fisher), 10% HPLC-grade acetonitrile (Fisher) before derivatization). Serum samples were mixed 1:1 with 6% sulfosalicylic acid (Fisher), incubated at room temperature for 5 min and centrifuged at 14,000 rcf for 8 min. All samples were spiked in with isotopes (3-C^13^ sodium lactate, 3-hydroxybutyric acid-d_4_ sodium, succinic acid-2,2,3,3-d_4_; 250 nM each), and then derivatized with 200 mM 3-nitrophenylhydrazine hydrochloride (Sigma-Aldrich; dissolved in 50/50 acetonitrile and water) and 120 mM 1-ethyl-3-(3-dimethylaminopropyl) carbodiimide hydrochloride (Pierce; dissolved in 47/47/6 acetonitrile, water and pyridine). Reactions were incubated at 37 °C for 45 min and diluted 1:50 in the extraction buffer for mass spectrometry (MS) analyses.

##### Data acquisition

Analysis used Agilent 6495c triple-quadrupole liquid chromatography–mass spectrometry (LC–MS) with the Agilent 1290 Infinity II UHPLC system and an ethylene-bridged hybrid C18 column (100 mm length, 2.1 mm inner diameter, 130 Å pore size, 1.7 μm particle size). Mobile phases were water (solvent A) and acetonitrile (solvent B) with 0.1% formic acid. Data were processed with Skyline v. 24.1 (ref. ^62^).

##### Data processing

Targeted metabolites were log_2_ transformed before analyses.

#### Immune protein measurements

##### Sample preparation

Fasting serum samples were prepared as per iPOP protocol^22^ and stored at −80 °C.

##### Data acquisition

Circulating cytokines were measured using a 76-plex Luminex antibody-conjugated bead capture assay (EMD Millipore), extensively characterized and benchmarked by Stanford Human Immune Monitoring Center^63^. Three panels were used: panel 1 (Milliplex HCYTMAG-60K-PX41 plus IL-18, IL-22), panel 2 (Milliplex HCP2MAG-62K-PX23 plus MIG) and panel 3 (9-plex from Milliplex HSP1MAG-63K-06 and HADCYMAG-61K-03). Samples were diluted 3-fold (panels 1 and 2) or 10-fold (panel 3). Diluted samples (25 µl) were mixed with antibody-linked magnetic beads in a 96-well plate and incubated overnight at 4 °C with shaking (500–600 rpm). Plates were washed twice with a wash buffer in an ELx405 washer (BioTek Instruments). Following a 1-h incubation at room temperature with biotinylated detection antibody, streptavidin-PE was added for 30 min with shaking. Plates were washed as described above, and PBS was added to wells for reading in a Luminex FlexMap3D with a lower bound of 50 beads per sample per cytokine. Custom Assay Chex control beads (Custom ACX) were added to all wells (Radix BioSolutions). Wells with <20 beads were excluded.

##### Data processing

Samples were randomly distributed between the plates, but longitudinal samples from the same participant were analyzed on the same plate. Due to sample volume limitations, single measurements were performed. Assay precision was controlled using Custom ACX control beads and inter-plate normalization. Inter-plate variability was corrected using median inter-plate ratios for four representative samples. Raw mean fluorescence intensity values were used for the analysis. Missing values (bead count <20) were imputed using the *k*-nearest neighbors (R ‘impute’ package v. 1.46.0). Cytokines were log_2_ transformed before downstream analyses.

#### Untargeted metabolomics and targeted lipidomics

##### Sample preparation

Plasma samples were thawed on ice and analyzed randomly as previously described^64^. Metabolites and complex lipids were extracted from 40 µl plasma using biphasic separation with cold methyl tert-butyl ether, methanol and water, and then reconstituted in 9:1 methanol–toluene. Each sample included 15 labeled metabolite internal standards (IS) for extraction efficiency and LC–MS performance monitoring, plus deuterated lipid IS (Sciex, catalog number 5040156, lot number LPISTDKIT-102b) for quantification. Quality controls included 3 reference plasma samples and 1 preparation blank.

##### Data acquisition

Plasma metabolites were analyzed using a broad-spectrum metabolomics platform with hydrophilic interaction chromatography (HILIC) and reverse-phase liquid chromatography (RPLC)–MS^65^. Complex lipids were quantified using targeted MS^64^.

For untargeted metabolomics by LC–MS, metabolic extracts underwent four analyses using HILIC and RPLC separation in positive and negative ionization modes. HILIC data were acquired on a Thermo Q Exactive HF mass spectrometer and RPLC on a Thermo Q Exactive, both operated in full MS scan mode. MS–MS data were collected on quality control (QC) samples consisting of an equimolar mixture of all study samples. HILIC used a ZIC-HILIC column (2.1 × 100 mm, 3.5 μm, 200 Å; Merck Millipore) and mobile phase solvents 10 mM ammonium acetate in 50/50 acetonitrile–water (A) and 10 mM ammonium acetate in 95/5 acetonitrile–water (B). RPLC used a Zorbax SBaq column (2.1 × 50 mm, 1.7 μm, 100 Å; Agilent Technologies) and mobile phase solvents consisting of 0.06% acetic acid in water (A) and 0.06% acetic acid in methanol (B). Quality assurance included (1) equilibration injections of 6- and 12-pool samples before running the sequence for RPLC and HILIC, respectively; (2) pool sample every 10 injections; and (3) verification of IS mass accuracy, retention time and peak shape.

For targeted lipidomics, Lipidyzer platform^64^ was used, with commercial flow injection having multiple reaction monitoring (MRM) and positive–negative ionization switches across 13 lipid classes using two methods (SelexION voltages on–off). QC involved (1) differential mobility spectrometry compensation voltage tuning using a set of lipid standards (catalog number 945 5040141, Sciex) after each cleaning, more than 24 h of idling or 3 days of consecutive use; (2) quick system suitability test (catalog number 50407, Sciex) before each batch to ensure an acceptable limit of detection for each lipid class; and (3) triplicate reference plasma sample (catalog number 948 4386703, Sciex) injection at the beginning of each batch.

##### Data processing

For untargeted metabolomics, data from each mode were processed using Progenesis QI v. 2.3 (Nonlinear Dynamics). Features from blanks and those with poor linearity following dilution in QC samples (Pearson correlation coefficient *r* < 0.6) were discarded. Only features present in >2/3 of samples were retained. Inter- and intra-batch variations were corrected using random forest normalization on QC samples^66^. Missing values were imputed from random low-value distributions. The merged dataset contained 5,681 metabolic features. Features were tentatively identified by matching fragmentation spectra and retention time to analytical-grade standards (metabolomics standards initiative (MSI) level 1) or MS–MS publicly available databases (MSI level 2). For duplicate annotations, only the feature with the highest median value was retained, yielding 467 metabolites. Abundances, reported as spectral counts, were log_2_ transformed.

For targeted lipidomics, Lipidyzer data were processed by Lipidomics Workflow Manager software, which calculates concentrations as analyte MRM/average intensity of the most structurally similar IS MRM multiplied by its concentration. Lipids detected in <2/3 of samples were discarded, with missing values imputed class-wise. The dataset contained 737 individual lipid species reported as nmol g^−1^ concentrations, and then log_2_ transformed before analyses.

### Statistical analyses

#### Clinical metadata

Clinical data included demographic, anthropomorphic, laboratory and cardiovascular functional and imaging data. Normal distribution was assessed using Shapiro–Wilk test. Student’s *t*-tests were used to compare normally distributed variables between HF and healthy cohorts at baseline, Mann–Whitney *U* tests were used for skewed distributions, and Fisher’s exact tests were used for categorical variables.

#### Metagenomic data

##### OMNIgene-GUT versus regular stool collection

A linear mixed model (LMM) evaluated collection method effects on paired samples (*n* = 18, total of 36 samples). Microbial features (alpha-diversity, taxonomic and functional features) were modeled as dependent variables with collection method as the independent variable. Beta-diversity used UniFrac (unweighted and weighted) and Bray–Curtis distance with PERMANOVA testing.

##### HF versus healthy comparison

LMMs accounted for age, sex, race, BMI, diet (fixed effects) and participant (random effect). Microbiome features (alpha-diversity, taxa, pathways and predicted metabolites^60^) were dependent variables, and categorical HF was the independent variable. Beta-diversity used weighted UniFrac and Bray–Curtis with PERMANOVA. The GSEA method^59^ for functional enrichment analysis estimated *P* values via 1,000 permutations with multiple testing correction.

##### Within HF cohort analysis

LMMs in the HF cohort (*n* = 59 patients, 86 total visits) included age, sex, race (‘white’ versus ‘other’), BMI, diet (fixed effects) and participant (random effect). Microbial features were dependent variables and clinical metrics were independent variables.

##### Medication analysis

Digoxin, diuretics and MRAs were analyzed using LMMs, with medication as the independent variable and microbiome features as dependent variables, adjusting for NYHA functional class.

##### Longitudinal data for the HF cohort

For 26 patients (52 total visits; for 1 individual who recorded 3 visits, we included only their baseline and last visit), beta-diversity used Bray–Curtis distance with PERMANOVA. Multivariate LMMs tested visit number effects. Between-visit clinical improvement was defined as an NYHA class decrease ≥1 level (*n* = 6), an LVEF increase ≥5% (*n* = 11 patients) or a KCCQ OSS increase ≥5 points (*n* = 8 patients). Binary improvement status was modeled as the independent variable, and microbiome features as dependent variables. Long-term clinical EMR data (*n* = 51 individuals, 75 total visits) analyzed NYHA and LVEF improvement using similar LMMs.

#### Multi-omic host data: cytokine, untargeted metabolomic and targeted metabolomic and lipidomic data

##### HF versus healthy

Due to a batch effect, only serum cytokines (76 features) and targeted metabolites (5 features) were compared between the HF (*n* = 58) and the healthy cohort (*n* = 48). PERMANOVA assessed global cytokine shifts. LMMs identified differentially abundant cytokines, adjusting for age, sex, race, BMI (fixed effects) and participant (random effect). Targeted metabolite models also included diet.

##### HF phenotype and severity

Multivariate LMMs adjusted for age, sex, race, BMI, diet (fixed effects) and participant (random effect), with multi-omic features as dependent variables and clinical variables as independent variables. For the volcano plot (Fig. 5a), log_2_(fold change) compared mean feature values between outcome groups. For the bile acid heat map (Fig. 5c), hierarchical clustering used Euclidian distance and average linkage, with coefficients normalized to median bile acid levels and *z*-score scaled. ‘ComplexHeatmap’ R package v.2.24.1 was used.

#### Microbiome–host interactions

##### Cytokine–microbiome correlations

Pairwise Spearman correlations between 100 most abundant microbial pathways and 76 cytokines were calculated for patients with HF (*n* = 58) with hierarchical clustering at both pathway and cytokine levels. The hierarchical cluster structure was then applied to correlations in the healthy cohort (*n* = 48). This identified five pathways for arginine and ornithine biosynthesis (ARGSYN-PWY: l-arginine biosynthesis I; ARGSYNBSUB-PWY: l-arginine biosynthesis II (acetyl cycle); PWY-5154: l-arginine biosynthesis III via *N*-acetyl-l-citrulline; PWY-7400: l-arginine biosynthesis IV (archaebacteria); and GLUTORN-PWY: l-ornithine biosynthesis I) with cohort-specific cytokine correlations. Aggregated pathway abundance was correlated (Spearman) with TNF (log_2_ transformed for visualization purposes) and categorized by median for C-reactive protein association.

##### *Bifidobacterium* association with host multi-omic biomarkers

*Bifidobacterium* genus and species relative abundances were modeled as independent variables in multivariate LMMs adjusting for demographics, BMI, diet (fixed effects) and participant (random effect).

#### Machine learning using multi-omic data for classification between chronic HF and the healthy state

This analysis excluded features that were available only for one of the cohorts (for example, NT-proBNP was available only for patients with chronic HF) or in which batch effects confounded HF and healthy cohorts (for example, metabolomics and lipidomics features, which were unavailable for healthy participants). This filtering left four data types for the analysis: laboratory tests (*n* = 53), cytokines (*n* = 76), microbial taxa (*n* = 553) and pathways (*n* = 306). Each of the 988 variables was first standardized (zero centered and scaled) among 132 individual data points (84 HF data points from 58 unique HF patients and 48 healthy participants), after which 80% of the samples were randomly selected from stratified groups as the training set. The remaining 20% of samples served as the holdout set. With healthy participants labeled as 0 and patients with chronic HF labeled as 1, Lasso and elastic-net models were fitted for regression on standardized multi-omic variables using coordinate descent as implemented in the R ‘glmnet’ and ‘caret’ packages. To derive feature importance, coefficients from the model trained with all four -omics types together were used.

#### Multiple hypothesis testing

The Benjamini–Hochberg method was used to correct for multiple hypotheses testing^67^, with *P*_adj_ < 0.2 considered statistically significant, provided that the corresponding nominal *P* value was <0.05. Analyses for which we did not perform Benjamini–Hochberg adjustment included PERMANOVA, cytokine–microbiome correlation (owing to its exploratory nature) and exometabolomics analyses.

### In vitro data

#### In vitro culturing of *Bifidobacterium* species

All *Bifidobacterium* culturing was performed in an anaerobic chamber. Individual colonies of *Bifidobacterium* species (well-studied type strains, as the study collection method did not preserve bacterial viability; Supplementary Table 14) were inoculated into Mega Media^68^ (Supplementary Table 14), grown at 37 °C for 24 h, diluted 1:100 into fresh medium and regrown for 24 h. Saturated cultures were centrifuged, and supernatants were used for targeted metabolite quantifications.

### Reporting summary

Further information on research design is available in the Nature Portfolio Reporting Summary linked to this article.

## Supplementary information

Supplementary Information Validation analysis and extended methods.

Reporting Summary

Supplementary Tables Supplementary Tables 1–15.

## Source data

Source Data Fig. 2 Gut microbiome features (alpha- and beta-diversity, taxa, pathways, GSEA) of HF and healthy cohorts.

Source Data Fig. 3 Correlations between gut microbiome pathways and host cytokines (HF and healthy cohorts).

Source Data Fig. 4 HF severity metrics, and gut microbiome features and association coefficients.

Source Data Fig. 5 HF severity metrics and host multi-omics data.

Source Data Fig. 6 HF improvement and gut microbiome features, and in vitro study targeted metabolomics data.

Source Data Extended Data Table 1 HF clinical variables.

Source Data Extended Data Fig. 1 Gut microbiome features by stool sample collection method (fresh frozen stool and OMNIgene-GUT kit).

Source Data Extended Data Fig. 2 Gut microbiome features (alpha- and beta-diversity, taxa, pathways) for the HF cohort and validation healthy cohort.

Source Data Extended Data Fig. 3 Additional gut microbiome features (alpha- and beta-diversity, taxa, pathways, GSEA) of HF and healthy cohorts.

Source Data Extended Data Fig. 4 Cytokines in HF and healthy cohorts, correlations between gut microbiome genera and cytokines, combined arginine pathways and cytokine levels in HF and healthy cohorts, combined arginine pathways and inflammatory marker CRP in the HF cohort.

Source Data Extended Data Fig. 5 HF severity metrics, select gut microbiome and metabolomics features.

Source Data Extended Data Fig. 6 HF improvement metrics, select gut microbiome and metabolomics features.

## Data Availability

Raw microbiome sequencing data were deposited in NCBI Sequence Read Archive under accession code PRJNA1266263 and are publicly available at http://www.ncbi.nlm.nih.gov/bioproject/1266263. Immune protein data were deposited in ImmPort (https://www.immport.org) under accession code SDY3093 and are publicly available at https://www.immport.org/shared/study/SDY3093. All processed data (including the clinical metadata and annotated microbiome, cytokine, metabolomic and lipidomic data) that support the findings of this study are publicly available via figshare at https://figshare.com/articles/dataset/Systolic_heart_failure_gut_microbiome_multi-omics_dataset/30422413 or https://doi.org/10.6084/m9.figshare.30422413 (ref. ^49^). Source data are provided with this paper.

## Extended data

**Extended Data Table 1:** Heart failure phenotypes for study participants at baseline

			Chronic HF (*n*=59) (mean (SD), median [IQR], or %)
Etiology of chronic HF		Idiopathic	44.1%
		Familial/genetic	35.6%
		Toxin-related	11.9%
		Other	8.4%
Functional status	NYHA functional class	I	33.9%
		II	42.4%
		III	20.3%
		IV	3.4%
	KCCQ scores	Overall summary score	82.3 [58.9, 92.2]
		Clinical summary score	91.4 [66.7, 100]
		Total symptom score	92.7 [75, 100]
Echocardiographic parameters		LVEF (%)	30.5 (11.6)
		LVEDD (mm)	63.3 (9.9)
		RV function	2 (0-4)
		RV size	0 (0-2)
		Estimated RVSP (mmHg)	36 [26, 51]
		Estimated RAP (mmHg)	5 [5, 10]

Abbreviations – HF: heart failure; SD: standard deviation; IQR: interquartile range; NYHA: New York Heart Association; KCCQ: Kansas City Cardiomyopathy Questionnaire; LVEF: left ventricular ejection fraction; LVEDD: left ventricular end-diastolic dimension; RV: right ventricular; RVSP: right ventricular systolic pressure; RAP: right atrial pressure. RV function and size are ordered categorical variables, with 0 representing normal RV function or size, and 5 severe RV dysfunction and enlargement.

Source data

## Code availability

All code was written in R v. 4.5.0 and MATLAB R2024b and is available via Bitbucket at https://bitbucket.org/kchuanglab/hf_gmb_2026/src/main/, GitHub at https://github.com/handuos/HF-GMB and Zenodo at https://doi.org/10.5281/zenodo.20756551 (ref. ^69^).
