# Supplementary material for: Gut microbiome shifts in chronic systolic heart failure are associated with disease severity and clinical improvement
Source: Nat Cardiovasc Res. 2026 Sep 24;5(10):1055–70. doi: 10.1038/s44161-026-00854-y (PMC13658019; doi:10.1038/s44161-026-00854-y)
Supplement: Supplementary file 1 [file 44161_2026_854_MOESM1_ESM.pdf]

nature cardiovascular research

Supplementary information

https://doi.org/10.1038/s44161-026-00854-y

Gut microbiome shifts in chronic systolic heart failure are associated with disease severity and clinical improvement

In the format provided by the authors and unedited

TABLE OF CONTENTS

Supplementary information 2

Validation analysis 2

Supplementary Information

Validation analysis

There was a significant sex imbalance between our original healthy cohort (*n*=50; a subset of the Integrated Personal Omics Profiling (iPOP) study cohort) and our chronic heart failure (HF) cohort (*n*=59), with women representing 52% of the healthy cohort and 22% of the HF cohort (Table 1). Even though our original multivariate analyses accounted for sex (Methods), we sought to more formally exclude the possibility of between-cohort sex imbalance driving our findings. Thus, we performed additional analyses using available metagenomic sequencing data from a larger healthy validation cohort from which our original healthy control cohort had been recruited.

For this analysis, we included 61 participants, of which 18 had a single visit (with 3 of those participants having 2 samples sequenced from the same visit that we treat as technical replicates); the analysis included 168 samples in total. These participants were selected from the larger available healthy cohort to bias toward men to more closely match the HF cohort. The final validation healthy cohort thus comprised of 45 men and 16 women (26%, compared to 22% in the HF cohort). Other demographics were reasonably matched as well (e.g., mean age 56.2 versus 52.3 years, and mostly Caucasian (68.9% versus 69.5%) compared to the HF cohort).

Of this validation cohort participants, 37 had sequencing data from the same visit in the original iPOP healthy cohort analysis, which allowed us to perform batch evaluation. This evaluation was pertinent as there were multiple differences in sample collection and processing between the three cohorts (Supplementary Table 15, shown below). Of the 857 total microbiome features (families, genera, species, and pathways), 385 (44.9%) were significantly affected by batch effects (p_{adj}<0.05), with most features depleted in the validation cohort (Supplementary Table 3). These effects were also reflected in alpha-diversity, which was significantly decreased in the validation cohort compared to the original healthy cohort. These changes were again expected given the

sample preparation protocol in the validation healthy cohort, which used ~1000-fold less DNA as input for library preparation, with the reads subsequently PCR amplified, thus biasing downstream results. Given this obvious batch effect, for the comparison between the HF and the validation healthy cohort we only included taxa and pathways that were not clearly affected by the batch effect (Supplementary Table 3), which included 42 families, 82 genera, 153 species, and 195 pathways.

Despite the above constraints, we were able to validate many of the key observations from our original analysis, including, among others, HF-associated depletion of *Bifidobacterium* and *Anaerostipes* and enrichment of *Prevotella* and *Sutterella* (Fig. S2a). In the functional analysis, we validated HF-associated loss of many short chain fatty acid (SCFA) biosynthetic pathways (including P124-PWY: *Bifidobacterium* shunt, CENTFERM-PWY: pyruvate fermentation to butanoate, and PWY-6590: superpathway of *Clostridium acetobutylicum* acidogenic fermentation). We also validated depletion of formaldehyde detoxification pathways in chronic HF, as well as enrichment of DTDPRHAMSYN-PWY (dTDP-L-rhamnose biosynthesis I), a key component of the lipopolysaccharide/endotoxin (Extended Data Fig. 2b). Notably, most of the validation cohort visits lacked curated dietary data, so our multivariate comparisons accounted for age, sex, race, and body mass index, but not diet.

	**Heart failure cohort (*n*=59, total of 86 samples)**	**Original healthy cohort (*n*=50)**	**Validation healthy cohort (*n*=61, total of 168 samples)**	**Notes**
Stool collection	OMNIgene-GUT kit	Fresh frozen, no preservative	Fresh frozen, no preservative	
DNA extraction kit	QIAamp PowerFecal DNA Kit	QIAamp PowerFecal DNA Kit	Zymobiomics 96 DNA Extraction Kit	Bias between Gram-positive and negative microbes
Required input DNA quantity	1 µg	1 µg	1 ng	Lower DNA input could result in lower alpha-diversity
Library prep kit	TruSeq DNA PCR-Free Low Throughput Library Prep kit	TruSeq DNA PCR-Free Low Throughput Library Prep kit	Illumina Nextera XT DNA library Prep kit	Additional bias and duplication introduced via PCR in the Nextera XT kit
Sequencing platform	HiSeqX	HiSeqX	NovaSeq	

Supplementary Table 15: Differences in sample collection and processing between the heart failure, original healthy, and validation healthy cohorts.
