# Supplementary material for: Gut microbiome shifts in chronic systolic heart failure are associated with disease severity and clinical improvement
Source: Nat Cardiovasc Res. 2026 Sep 24;5(10):1055–70. doi: 10.1038/s44161-026-00854-y (PMC13658019; doi:10.1038/s44161-026-00854-y)
Supplement: Supplementary file 2 [file 44161_2026_854_MOESM2_ESM.pdf]

nature portfolio

Corresponding author(s): Michael Snyder

Last updated by author(s): 6/18/2026

# Reporting Summary

Nature Portfolio wishes to improve the reproducibility of the work that we publish. This form provides structure for consistency and transparency in reporting. For further information on Nature Portfolio policies, see our Editorial Policies and the Editorial Policy Checklist.

## Statistics

For all statistical analyses, confirm that the following items are present in the figure legend, table legend, main text, or Methods section.

| n/a | Confirmed | |
|---|---|---|
| ☐ | ☒ | The exact sample size ($n$) for each experimental group/condition, given as a discrete number and unit of measurement |
| ☐ | ☒ | A statement on whether measurements were taken from distinct samples or whether the same sample was measured repeatedly |
| ☐ | ☒ | The statistical test(s) used AND whether they are one- or two-sided<br>*Only common tests should be described solely by name; describe more complex techniques in the Methods section.* |
| ☐ | ☒ | A description of all covariates tested |
| ☐ | ☒ | A description of any assumptions or corrections, such as tests of normality and adjustment for multiple comparisons |
| ☐ | ☒ | A full description of the statistical parameters including central tendency (e.g. means) or other basic estimates (e.g. regression coefficient) AND variation (e.g. standard deviation) or associated estimates of uncertainty (e.g. confidence intervals) |
| ☐ | ☒ | For null hypothesis testing, the test statistic (e.g. $F$, $t$, $r$) with confidence intervals, effect sizes, degrees of freedom and $P$ value noted<br>*Give P values as exact values whenever suitable.* |
| ☒ | ☐ | For Bayesian analysis, information on the choice of priors and Markov chain Monte Carlo settings |
| ☒ | ☐ | For hierarchical and complex designs, identification of the appropriate level for tests and full reporting of outcomes |
| ☒ | ☐ | Estimates of effect sizes (e.g. Cohen's $d$, Pearson's $r$), indicating how they were calculated |

*Our web collection on statistics for biologists contains articles on many of the points above.*

## Software and code

Policy information about availability of computer code

| | |
|---|---|
| Data collection | No software was used for data collection. |
| Data analysis | For metagenomics data, adapters and low-quality bases were trimmed using Trimmomatic with options "ILLUMINACLIP:TruSeq3-SE.fa:2:30:10 SLIDINGWINDOW:4:20 MINLEN:50". Reads from human cells were removed using KneadData v0.7.4 against the human genome (hg37 reference database). Exact duplicate reads were removed with clumpify (BBtools). The results were further analyzed using Bracken v2.6, HUMAnN v3.0, and MelonnPan v0.99.0. Further data analyses were performed with R 4.5.0 and MATLAB R2024b. The code is available at https://bitbucket.org/kchuanglab/hf_gmb_2026/src/main/ (Bitbucket), https://github.com/handuos/HF-GMB (GitHub), and https://doi.org/10.5281/zenodo.20756551 (Zenodo). |

For manuscripts utilizing custom algorithms or software that are central to the research but not yet described in published literature, software must be made available to editors and reviewers. We strongly encourage code deposition in a community repository (e.g. GitHub). See the Nature Portfolio guidelines for submitting code & software for further information.

## Data

Policy information about availability of data

All manuscripts must include a data availability statement. This statement should provide the following information, where applicable:

- Accession codes, unique identifiers, or web links for publicly available datasets
- A description of any restrictions on data availability
- For clinical datasets or third party data, please ensure that the statement adheres to our policy

Raw microbiome sequencing data were deposited in NCBI Sequence Read Archive under accession number PRJNA1266263 and are available at http://www.ncbi.nlm.nih.gov/bioproject/1266263.
Immune protein data were deposited in the ImmPort (https://www.immport.org) under accession number SDY3093 and are available at https://www.immport.org/shared/study/SDY3093.
All processed data, including the clinical metadata and annotated microbiome, cytokine, metabolomic, and lipidomic data, that support the findings of this study are available in the figshare repository at: https://figshare.com/articles/dataset/Systolic_heart_failure_gut_microbiome_multi-omics_dataset/30422413, or at DOI: https://doi.org/10.6084/m9.figshare.30422413.

## Research involving human participants, their data, or biological material

Policy information about studies with human participants or human data. See also policy information about sex, gender (identity/presentation), and sexual orientation and race, ethnicity and racism.

| | |
|---|---|
| Reporting on sex and gender | We collected sex information of all participants in the study. We did not collect gender information. The findings apply to male and female. |
| Reporting on race, ethnicity, or other socially relevant groupings | Participant race and ethnicity data were based on self-reported information. The participant-recruitment process was conducted predominantly in the San Francisco Bay Area, with inclusion of patients from other parts of California as they received specialty heart failure care at our institution. Owing to the demographic characteristics of this region, the resulting cohort predominantly consisted of individuals identifying as Caucasian and Asian. Recognizing the potential influence of race and ethnicity on the study findings, this variable was always incorporated as a variable in our statistical models. When we examined the relationship between the heart failure severity and the gut microbiome and host multi-omic signatures, given smaller sample size, we transformed this variable from a categorical variable with 6 different levels to a binary variable (white vs non-white). |
| Population characteristics | Our study included 59 adults with chronic systolic heart failure from non-ischemic cardiomyopathy (mean age 52.3 years, 22% female, 69.5% white, BMI 27.4 kg/m2) and 50 healthy controls previously recruited for the iPOP study (mean age 55.3 years, 52% female, 66% white, BMI 28.1 kg/m2). |
| Recruitment | Participants with heart failure were referred to the study by their treating heart failure cardiologist, after which a member of the research team discussed the details of the study after which participants signed the written informed consent. Participants from the healthy cohort had already been recruited for the iPOP study. There was no compensation for study participation. |
| Ethics oversight | The study protocol and clinical investigation were approved by the Stanford University School of Medicine Human Research Protection Office (Institutional Review Board # 44967). Participants provided informed consent to participate in the study. |

Note that full information on the approval of the study protocol must also be provided in the manuscript.

# Field-specific reporting

Please select the one below that is the best fit for your research. If you are not sure, read the appropriate sections before making your selection.

☒ Life sciences ☐ Behavioural & social sciences ☐ Ecological, evolutionary & environmental sciences

For a reference copy of the document with all sections, see nature.com/documents/nr-reporting-summary-flat.pdf

# Life sciences study design

All studies must disclose on these points even when the disclosure is negative.

| | |
|---|---|
| Sample size | In this study, we did not conduct a formal power calculation due to the nature of our heart failure patient cohort, which was established through convenience sampling. However, prior research investigating the gut microbiome in heart failure patients demonstrated the ability to identify statistically significant differences compared to control groups with fewer than 30 participants per group. Furthermore, those studies employed microbiome profiling techniques of lower resolution than those that we utilized, in addition to being cross-sectional in design, whereas our approach incorporated longitudinal sampling. Consequently, we made the deliberate decision to recruit at least 50 participants in the heart failure cohort to enhance our capability to detect specific gut microbiome and multi-omic signatures related to varying stages of heart failure severity. The final sample sizes achieved in this study—comprising 59 patients with heart failure and 50 healthy controls—enabled us to identify significant differences in gut microbiome composition between the cohorts, as well as to characterize microbiome signatures associated with heart failure severity and the temporal dynamics of the disease. |

| | |
|---|---|
| Data exclusions | No data were excluded. |
| Replication | Our cross-sectional results were validated with the longitudinal measurements. Moreover, we make all of our data freely available so they can be easily verified for reproducibility. |
| Randomization | Randomization was not applicable to the study |
| Blinding | Blinding was not applicable to the study |

# Reporting for specific materials, systems and methods

We require information from authors about some types of materials, experimental systems and methods used in many studies. Here, indicate whether each material, system or method listed is relevant to your study. If you are not sure if a list item applies to your research, read the appropriate section before selecting a response.

## Materials & experimental systems

| n/a | Involved in the study | |
|---|---|---|
| ☒ | ☐ | Antibodies |
| ☒ | ☐ | Eukaryotic cell lines |
| ☒ | ☐ | Palaeontology and archaeology |
| ☒ | ☐ | Animals and other organisms |
| ☐ | ☒ | Clinical data |
| ☒ | ☐ | Dual use research of concern |
| ☒ | ☐ | Plants |

## Methods

| n/a | Involved in the study | |
|---|---|---|
| ☒ | ☐ | ChIP-seq |
| ☒ | ☐ | Flow cytometry |
| ☒ | ☐ | MRI-based neuroimaging |

## Clinical data

Policy information about clinical studies

All manuscripts should comply with the ICMJE guidelines for publication of clinical research and a completed CONSORT checklist must be included with all submissions.

| | |
|---|---|
| Clinical trial registration | *Provide the trial registration number from ClinicalTrials.gov or an equivalent agency.* |
| Study protocol | *Note where the full trial protocol can be accessed OR if not available, explain why.* |
| Data collection | *Describe the settings and locales of data collection, noting the time periods of recruitment and data collection.* |
| Outcomes | *Describe how you pre-defined primary and secondary outcome measures and how you assessed these measures.* |

## Plants

| | |
|---|---|
| Seed stocks | *Report on the source of all seed stocks or other plant material used. If applicable, state the seed stock centre and catalogue number. If plant specimens were collected from the field, describe the collection location, date and sampling procedures.* |
| Novel plant genotypes | *Describe the methods by which all novel plant genotypes were produced. This includes those generated by transgenic approaches, gene editing, chemical/radiation-based mutagenesis and hybridization. For transgenic lines, describe the transformation method, the number of independent lines analyzed and the generation upon which experiments were performed. For gene-edited lines, describe the editor used, the endogenous sequence targeted for editing, the targeting guide RNA sequence (if applicable) and how the editor was applied.* |
| Authentication | *Describe any authentication procedures for each seed stock used or novel genotype generated. Describe any experiments used to assess the effect of a mutation and, where applicable, how potential secondary effects (e.g. second site T-DNA insertions, mosiacism, off-target gene editing) were examined.* |
